# Valorisation of Freshwater Fish By‐Products: A Review on Extraction Technologies, Stability, Functional Food Applications and Prospects

**DOI:** 10.1155/ijfo/7432242

**Published:** 2026-09-23

**Authors:** Tan Yi-Li, Abu Bakar Asyrul-Izhar, Nurul Izzah Khalid, Rahmi Nurdiani, Nurul Huda, Yongyong Li, Mohammad Rashedi Ismail-Fitry

**Affiliations:** ^1^ Department of Food Technology, Faculty of Food Science and Technology, Universiti Putra Malaysia, Serdang, Malaysia, upm.edu.my; ^2^ Faculty of Fisheries and Marine Science, Universitas Brawijaya, Malang, East Java, Indonesia, ub.ac.id; ^3^ Postgraduate School, Universitas Brawijaya, Malang, East Java, Indonesia, ub.ac.id; ^4^ College of Food Science and Engineering, Ningbo University, Ningbo, China, nbu.edu.cn; ^5^ Zhejiang Key Laboratory of Intelligent Food Logistic and Processing, Zhejiang-Malaysia Joint Research Laboratory for Agricultural Product Processing and Nutrition, Ningbo University, Ningbo, China, nbu.edu.cn; ^6^ Halal Products Research Institute, Universiti Putra Malaysia, Serdang, Malaysia, upm.edu.my

**Keywords:** bioactive compounds, extraction technologies, freshwater fish by-product, functional foods, valorisation

## Abstract

The valorisation of freshwater fish by‐products represents an emerging opportunity to reduce waste and enhance resource efficiency in the aquatic processing sector. These by‐products, including skin, bones, scales, viscera and heads, are rich in nutritionally valuable components such as proteins, collagen, gelatine, peptides and fish oils with bioactive potential. This review provides an overview of the composition and functional properties of these compounds and discusses current extraction technologies to maximise their recovery. Conventional methods, such as acid or alkaline extraction, remain widely used, but many compromise product quality and generate chemical waste. In contrast, enzymatic and assisted extraction methods, including ultrasound‐, microwave‐ and hydrothermal‐assisted processes, have demonstrated improved yield, hydrolysis efficiency and bioactivity, with lower environmental impact. Nonetheless, challenges in cost, scalability and process optimisation remain. The current review also highlights factors influencing the stability and storage of extracted compounds, including temperature, pH, oxidation and enzymatic degradation, which can affect bioactivity and shelf life. Stabilisation approaches such as freeze‐drying, encapsulation and active packaging have shown promise in preserving the structural integrity and viability of freshwater fish by‐products, enabling their transformation into high‐value ingredients for food, nutraceutical and biomedical applications, and supporting a more sustainable fisheries sector.

## 1. Introduction

Freshwater fish constitute a vital component of global food systems, representing a significant share of aquaculture production and serving as a critical source of protein for millions. According to the Food and Agriculture Organization (FAO), freshwater species accounted for 44% of total global finfish production and 33% of total aquatic animal production in 2022 [1]. Notably, over 83% of freshwater fish were farmed, indicating an increasing reliance on aquaculture to meet growing protein demands. Although aquaculture enhances food security and economic development, it also raises pressing concerns about sustainability, including high resource inputs, feed inefficiencies and the generation of substantial waste throughout the value chain, from production to processing and consumption [2–5].

Globally, the fish processing industry generates vast quantities of solid waste. Previous research showed that fish‐filleting, salting and smoking account for 50%–70% of total biomass waste, estimated at 3.17 million tonnes annually, whereas fish canning generates an additional 1.5 million tonnes of waste [6]. The amount of fish loss and waste in the supply chain has increased drastically to 23.8 million tonnes by mid‐2025 [7]. These by‐products, including heads, bones, scales, skin and viscera, are often undervalued or improperly discarded, leading to environmental degradation and public health risks due to pollution and pest breeding grounds [3, 4].

Recent advances in biowaste valorisation have shown that fish‐derived residues can be converted into high‐value products [8]. For instance, fish bones and scales are increasingly used to synthesise hydroxyapatite for biomedical and environmental applications [9]. A previous study extracted gelatine from freshwater fish scales into gelatine sheets or powder using an acid‐assisted extraction method [10]. Antu et al. [11] applied chemical and thermal treatments to pangas fish bones to obtain bone mineral powder for use in mineral supplements. Similarly, tilapia and catfish have been processed into nano‐calcium powder for application in calcium supplements [12]. Proper valorisation of fish by‐products holds promise for circular economy approaches, offering sustainable alternatives to synthetic ingredients while reducing ecological footprints.

In parallel, global demand for functional foods—those that provide health benefits beyond basic nutrition—has grown rapidly, driven by rising consumer awareness, ageing populations and the prevalence of diet‐related diseases. Recently, there has been a surge in interest in functional foods fortified with bioactive compounds such as omega‐3 fatty acids, peptides and collagen, many of which can be derived from fish by‐products [13]. This aligns with biodiversity‐based approaches to nutrition and supports the Sustainable Development Goals by linking health promotion with environmental responsibility [14].

Several reviews have previously addressed fish waste and by‐products. For instance, Jimenez‐Champi et al. [15] reviewed marine fishery by‐products, focusing on the nutritional content and bioactive compounds derived from various types of by‐products that can be transformed into valuable sources. The authors also discussed the application of these bioactive compounds in food systems, such as active food packaging films, chitosan‐enriched meat products and yoghurt fortified with fish oil, as well as technologies including extrusion and hydroextraction processes. In their review, Benkhoud et al. [16] reported processing sardine gills and viscera into wheat flour–based chips with potential antidiabetic and hepatoprotective properties. Yin et al. [17] focused on the extraction of calcium from marine fish heads, bones and scales. Likewise, Akhtar Hussain and Yasmin [18] concentrated on the production and application of value‐added products. Collectively, these researchers summarised that marine fish waste‐derived materials hold broad potential for conversion into value‐added commodities, including fish meal and oil for aquaculture and agriculture, collagen and gelatine for food and pharmaceutical applications, biofertilisers for soil improvement, biodiesel for renewable energy and bioactive peptides and enzymes with functional and therapeutic properties.

Although many valorisation strategies developed for marine fish by‐products are potentially transferable to freshwater species, important compositional and processing differences justify separate consideration. Freshwater and marine fish differ in lipid composition, odour‐forming compounds, collagen characteristics and environmental adaptation, which may influence extraction efficiency, product stability and functional properties [19, 20]. Marine fish generally contain higher levels of trimethylamine oxide (TMAO), contributing to stronger fishy odours during processing, whereas freshwater fish exhibit different flavour profiles and more variable fatty acid compositions depending on species and farming conditions [21, 22]. In addition, variations in collagen thermal stability and amino acid composition of fish species from different aquatic environments may affect gelatine and collagen extraction behaviour [23]. Therefore, optimising extraction technologies and product applications may require freshwater‐specific approaches, despite similarities in overall valorisation concepts.

**Figure 1:**
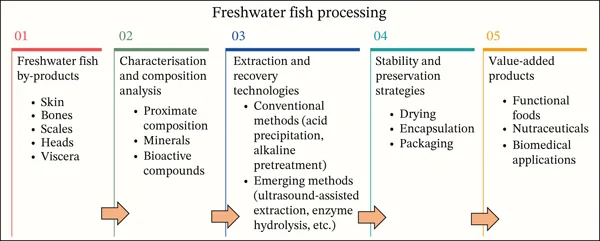
Schematic diagram summarising the valorisation chain of freshwater fish by‐products.

In addition, limited reviews are available on freshwater fish by‐products; therefore, the present review is aimed at exploring their sustainable valorisation by analysing existing extraction technologies, their impact on product stability and their applications in the development of functional foods. By identifying key challenges and opportunities across the valorisation chain, this review seeks to contribute to a more resource‐efficient and sustainable freshwater aquaculture system. The conceptual framework guiding this review is illustrated in Figure 1. The diagram illustrates the progression from freshwater fish by‐products to value‐added products through characterisation, extraction and stabilisation strategies within a framework.

## 2. Overview of Freshwater Fish By‐Products

### 2.1. Types and Sources of Freshwater Fish By‐Products

Freshwater aquaculture and capture fisheries across South East Asia are dominated by species such as freshwater catfish (*Clarias* spp.), snakehead fish (*Channa* spp.), gourami (*Osphronemus goramy*), iridescent shark (*Pangasionodon hypophthalmus*), tilapia (*Oreochromis* spp.), climbing perch (*Anabas testudineus*), hampala barb (*Hampala macrolepidota*), mahseer (*Tor* spp.), Hoven′s carp (*Leptobarbus hoevenii*), Java barb (*Barbonymus gonionotus*) and pacu (*Piaractus brachypomus*). These species represent a major share of regional production and wet‐market supply [24]. Almost 90% of freshwater fish species are found in eight orders: carps, minnows and loaches (Cyprinodontiformes), gobies (Gobiiformes), perch‐like fishes (Perciformes), bettas and gouramis (Anabantiformes) [25]. The Hull International Fisheries Institute and the FAO found that 50% of global freshwater fish catch originates from seven river basins: the Mekong, Nile, Irrawaddy, Yangtze, Brahmaputra, Amazon and Ganges.

Processing operations such as filleting, gutting and trimming generate substantial quantities of by‐products, representing a valuable yet underutilised resource. Global fish production is projected to reach 201 million tonnes by 2030, with aquaculture contributing an increasingly dominant share, highlighting the scale of by‐product generation and the urgency for sustainable valorisation [8]. Table 1 summarises the morphological characteristics and major by‐product fractions of selected freshwater fish species.

**Table 1:** Morphological characteristics and body composition of selected freshwater fish species.

Fish species	Average length (cm)	Average weight (kg)	Head (%)	Bone (%)	Skin (%)	Viscera (%)	References
Freshwater catfish (*Clarias* spp.)	51–195	0.6–2.5	29.1	14.33	4.7	6.89	Brummett [26]; Ferosekhan et al. [27]; Khan et al. [28]; Kord et al. [29]
Snakehead fish (*Channa* spp.)	33–45	~0.45	20.06	9%–15%	3.91	4.19	Herpandi et al. [30]; Sari and Rohma [31]
Gourami (*Osphronemus goramy*)	16.4	0.16	20.07	16.03	7.09	8.41	Syandri et al. [10]
Irisdescent shark (*Pangasionodon hypophthalmus*)	43	1.25	N/A	N/A	8.0	8.0	Islami et al. [32]
Tilapia (*Oreochromis niloticus* spp.)	17.5–23.6	0.5–0.6	21.35	14.63	5.57	8.88	Khalid et al. [33]; Relekar et al. [34]; Syandri et al. [10]
Silver carp (*Hypophthalmichthys molitrix*)	13–58.5	0.9–1.2	27.5	23	N/A	7.2	Ferguson et al. [35]; Relekar et al. [34]; Sahu et al. [36]; Zhu et al. [37]
Climbing perch (*Anabas testudineus*)	7.40–14.50	0.0079–0.064	N/A	N/A	N/A	N/A	Mawa et al. [38]
Hampala barb (*Hampala macrolepidota*)	15.7–44.5	0.5–3.0	N/A	N/A	N/A	N/A	Herawati et al. [39]; Makmur et al. [40]
Mahseer (*Tor* spp.)	12–46	0.005	N/A	N/A	N/A	0.05–0.09	Ibrahim et al. [41]; Kamarudin et al. [42]
Hoven′s carp (*Leptobarbus hoevenii*)	7.1–11.7	0.3–3.5	N/A	N/A	N/A	1.03–12.28	Srithongthum et al. [43]; Mohd Khairul Amri et al. [44]
Java barb (*Barbonymus gonionotus*)	8.9–28.5	0.2–1.5	0.7	4.6–8.5	2.6	20.9	Sahu et al. [45]; Tamsil and Hasnidar [46]
Pacu (*Piaractus brachypomus*)	24.34	1–20	N/A	N/A	N/A	N/A	Murthy et al. [47]

*Note:* N/A means not available.

Freshwater fish by‐products include heads, viscera, scales, skin, tails, bones, offal and blood, which may account for 30%–40% of total fish biomass and are often discarded or downgraded to low‐value uses [48, 49]. They are typically generated at primary processing centres, wet markets and small‐scale filleting plants, where limited processing infrastructure results in improper disposal or conversion into low‐value products, such as animal feed or compost. Such practices can cause environmental pollution and public health concerns, especially in regions lacking adequate waste treatment systems [50]. Understanding the types and sources of freshwater species is essential, as effective valorisation strategies rely on consistent and well‐characterised feedstock. The subsequent section discusses the nutritional potential of these by‐products as valuable sources of functional food ingredients.

### 2.2. Nutritional Composition and Bioactive Compounds of Freshwater Fish By‐Products

Building on the by‐products described above, this section summarises their nutritional composition and bioactive potential. Freshwater fish by‐products retain substantial nutritional and functional value, including proteins, lipids, minerals and bioactive compounds, making them promising feedstocks for sustainable food, nutraceutical and biomedical applications. According to FAO [51], typical by‐product processing yields include heads (9%–12%), offal (12%–18%), skin (1%–3%), bones (9%–15%) and scales (5%), most of which are discarded despite their high nutrient density. These materials exhibit antioxidant, antimicrobial, antihypertensive and mineral‐chelating properties [15, 52]. Table 2 shows the biochemical composition of several freshwater fish by‐products analysed in various studies.

**Table 2:** Types of fish by‐products and their biochemical composition.

Fish species	By‐product type	Protein (%)	Fat (%)	Ash (%)	Moisture (%)	References
African catfish (*Clarias gariepinus*)	Head, bones, skin and fins	12.27	15.06	3.87	68.84	Adnan et al. [53]
	Head	48.42	11.48	32.88	6.65	Kord et al. [29]
	Collagen (from skin)	89.09	N/A	N/A	N/A	Kord et al. [29]
	Gelatin (from skin)	77.40	N/A	N/A	N/A	Kord et al. [29]

Striped catfish (*Pangasianodon hypophthalmus*)	Head, bones, skin and fins	13.76	17.65	5.21	55.85	Adnan et al. [53]

Tilapia (*Oreochromis mossambicus*)	Head, bones, skin and fins	12.59	3.36	4.72	70.11	Adnan et al. [53]

Nile tilapia (*Oreochromis niloticus*)	Backbone flour	55.41	N/A	28.38	3.53	Corrêa et al. [54]
	Head flour	39.89	N/A	36.85	3.18	Corrêa et al. [54]
	Head (oil source)	12.3	10.9	9.5	67.3	Moraes et al. [55]
	Skin (leather)	29.08	1.96	1.82	67.14	Franco et al. [56]
	Viscera	14.01	20.00	4.75	60.62	Purnamayati et al. [57]

Snakehead (*Channa striata*)	Head, bones, fins and tail	17.09–19.02	2.45–4.26	1.30–30.18	7.05–16.42	Sasongko et al. [58]

Common carp (*Cyprinus carpio*)	Scales	34.57 (wwb)	0.45 (wwb)	5.94 (wwb)	36.00 (wwb)	Oktarlina et al. [59]

Silver carp (*Hypophthalmichthys molitrix*)	Scales	48.08	0.77	31.68	16.71	Wu et al. [60]

*Note:* N/A means not available.

Notably, by‐products from striped catfish (*P*. *hypophthalmus*), African catfish (*Clarias gariepinus*), channel catfish (*Ictalurus punctatus*) and tilapia (*Oreochromis* spp.) exhibit protein contents of 12%–55%, fat of 3%–20%, ash of 4%–37% and moisture of 3–%70%, depending on tissue type and processing method, confirming their suitability for protein, lipid and mineral recovery [53, 54, 56, 29, 55].

Viscera, which account for 16%–20% of total fish mass, yield 50%–70% protein when processed into hydrolysates and are rich in digestive enzymes, lipids and antioxidant peptides [53, 57, 61–63]. Valorisation approaches such as enzymatic hydrolysis and supercritical carbon dioxide (CO_2_) extraction convert viscera into fish protein hydrolysates (FPHs) and oils rich in omega‐3 fatty acids [15, 64].

Fish bones are particularly valuable due to their high mineral content, especially calcium and phosphorus, as well as collagen and bioactive peptides. Advanced processing techniques can yield calcium‐rich powders suitable for food fortification and therapeutic applications [65]. Protein content in freshwater fish bone powders typically ranges from 18% to 27%, with calcium levels up to 793 mg/100 g [66–68]. Such powders have been successfully incorporated into bakery goods, pasta and beverages, serving as a functional, accessible calcium source, particularly beneficial for individuals with lactose intolerance or dietary restrictions. The mineral profile of fish bones includes essential nutrients such as calcium (Ca), phosphorus (P) and iron (Fe), with studies noting mineral content ranging from 21% to 57% and organic matter between 20% and 30% [21].

Other structural by‐products, including heads, fins, tails and scales, also demonstrate high protein (17%–48%) and mineral content (2%–32% ash), with collagen extracted as Type 1 fibrous proteins retaining triple‐helical integrity [59, 60]. Collagen hydrolysates exhibit antioxidant, antihypertensive and mineral‐binding properties, supporting their use in functional foods, nutraceuticals, cosmetics and biomedical applications [52].

Overall, these by‐products represent efficient sources of protein, lipids, minerals and bioactive proteins, with multiple valorisation pathways enabling their incorporation into foods, supplements and therapeutic formulations (Table 2).

### 2.3. Potential and Value‐Added Products From Freshwater Fish By‐Products

The by‐product fractions and composition profiles described above determine which value‐added products can realistically be recovered from each tissue. At present, much of this material is diverted to low‐value outlets such as fishmeal, silage, compost or direct animal feed [50], where returns are marginal, and the nutritional potential outlined in Section 2.2 remains largely unrealised. A growing body of work on freshwater species demonstrates that the same fractions can instead be converted into defined products of substantially higher value. This section outlines the main product categories reported for freshwater fish by‐products, the tissues from which they are conventionally obtained and their principal applications. Products are grouped by compound class so that the compositional data in Table 2 can be read directly against the product streams they support; the extraction routes themselves are addressed in Section 3, and food incorporation trials in Section 5.

Collagen is the product most closely associated with skin and scales, the two fractions richest in structural protein. Type I collagen has been recovered from the skin of catfish, clown knifefish and tilapia [69], from the scales of silver carp [60] and common carp [59], and from the skin of African catfish, where the isolated collagen reached 89.09% protein [29]. Collagen is conventionally supplied as a hydrolysed powder for nutraceutical and cosmetic use, exploiting its amino acid profile and its reported antioxidant, antihypertensive and mineral‐binding activities [52]. Finer particle‐size formats extend the range of applications: Microcollagen has been prepared from carp scale waste [59], whereas nano‐collagen produced from catfish skin was formulated into a topical cream that accelerated the healing of second‐degree burns in rabbits, reducing wound size and promoting vascularisation relative to untreated controls [70]. These findings indicate biomedical potential, although the number of nano‐collagen formulations approved for commercial use remains small, and questions of large‐scale production, stability and clinical efficacy are unresolved [71].

Gelatine, the partially hydrolysed derivative of collagen, is obtained from the same tissues—skin, scales and bone—and is the most commercially established product in this group. Gelatine has been prepared from African catfish skin at 77.40% protein [29], from freshwater fish scales using *Averrhoa bilimbi* as a natural acidulant to give gelatine in sheet or powder form [10], and from mixed catfish and tilapia by‐products by hydrothermal treatment, giving a product with gel strength and viscosity considered acceptable for industrial application [53]. Its applications span gelling, thickening and stabilising functions in confectionery, dairy and meat systems, encapsulating matrices, and edible films and coatings [72, 73]. Fish gelatine has additionally been reported to act as a dietary antihypertensive agent and to support wound repair and tissue regeneration [74]. A further commercial driver is its position as an alternative to bovine and porcine gelatine, addressing religious dietary requirements and consumer preferences in Asian and Middle Eastern markets [75].

FPHs and bioactive peptides are produced from fractions too fragmented or heterogeneous for structural protein recovery, including trimmings, frames, heads and viscera. Hydrolysates prepared from Nile perch trimmings using combined endo‐ and exopeptidase systems yielded low‐molecular‐weight peptides with over 60% angiotensin‐converting enzyme inhibition at 5 mg/mL, indicating antihypertensive potential. However, antidiabetic and anti‐Alzheimer activities were low [76]. Hydrolysates from trout frames showed improved solubility, emulsifying activity and foaming capacity alongside antioxidant activity, demonstrating that FPH offer techno‐functional as well as physiological value [77]. Typical applications include protein‐fortifying ingredients in beverages, soups and nutraceutical powders. The principal constraint is sensory rather than nutritional: Peptidase action generates short‐chain peptides that may be bitter [78], and hydrolysates can precipitate or develop off‐flavours in beverage systems unless encapsulated or flavour‐masked.

Fish protein concentrate (FPC) represents a simpler, lower specification protein product obtained by solvent removal of lipid from protein‐rich tissue. FPC produced from tilapia using isopropyl alcohol met the FAO Category C specification (protein below 60%, fat above 3%) and was recovered as a dry powder suitable for use as a fortifying ingredient [79]. Although its functional value is lower than that of enzymatically produced hydrolysates, FPC requires less specialised equipment and remains relevant where processing infrastructure is limited.

Fish oil is recovered from the highest lipid fractions, principally viscera and heads; Table 2 shows tilapia viscera at 20% fat and striped catfish by‐products at 17.65%. Oil extraction from Nile tilapia viscera has been optimised by response surface methodology [57], and oil has been characterised from three freshwater catfish species with differing chemical quality profiles [80]. Hydrothermal treatment of mixed catfish and tilapia by‐products recovered oil rich in omega‐9, omega‐6 and omega‐3 fatty acids concurrently with gelatine, illustrating that a single processing stream can yield more than one marketable product [53]. Applications include omega‐3 supplements and encapsulated oil powders for functional food formulation. Freshwater fish oils contain eicosapentaenoic and docosahexaenoic acids (DHAs) at lower and more variable concentrations than oily marine species [81], so positioning as a general‐purpose lipid ingredient is more realistic than positioning as a high‐potency omega‐3 source. Lower grade fractions also have non‐food outlets, as Nile tilapia processing waste has been evaluated as a biodiesel feedstock [55].

Bones and scales support a family of mineral products distinguished mainly by the degree of processing applied. The least processed is bone or head flour, produced by drying and milling: backbone and head flours from Nile tilapia contained 55.41% and 39.89% protein with 28.38% and 36.85% ash, respectively, and were prepared for inclusion in instant food products [54]. More refined is nano‐calcium, obtained by acid‐alkali hydrolysis and neutralisation, with tilapia bone giving a finer particle size (87.37 nm) than catfish [12]; mineral powders have similarly been recovered from pangas bone for use as supplements [11]. At the highest level of processing, hydroxyapatite has been synthesised from armoured catfish bone by thermal calcination and used as a filler in chitosan‐based membranes, with potential in bone tissue engineering and environmental remediation [82], whereas hydroxyapatite‐collagen nanocomposites have been designed for biomedical use [83]. The commercial rationale is favourable, as raw fish bone contains over 10% calcium by fat‐free dry weight, comparable to conventional sources such as calcium citrate, lactate, gluconate and acetate [65]. Mineral‐fortification trials in bakery, snack and noodle products are discussed in Section 5.

Beyond food and nutraceutical uses, several fractions support industrial applications that can absorb material that fails food‐grade specifications. Tilapia skin has been evaluated as a leather substrate, with its histological structure, composition and tensile resistance compared against pacu and tambaqui [56]; bone‐derived hydroxyapatite has been applied in environmental remediation [9]; and lipid fractions unsuitable for food use can be directed to biodiesel production [55].

Taken together, these examples show that a single freshwater fish processing stream can generate several products at different value tiers—collagen and gelatine from skin and scales, hydrolysates from trimmings and frames, oil from viscera and heads and mineral products from bone—rather than a single low‐value output. This multiplicity is central to the economic case for valorisation, as it allows the fixed costs of by‐product collection, handling and cold‐chain management to be distributed across several revenue streams. The extent to which each product can be realised in practice depends on the extraction technology applied, which is examined in the following section.

## 3. Extraction Technologies

The valorisation of freshwater fish by‐products requires efficient extraction technologies to isolate and preserve valuable bioactive compounds such as collagen, gelatine and fish oil. Extraction methods can be broadly categorised as conventional or advanced, each with distinct advantages and limitations. Conventional methods, including acid precipitation and alkaline pretreatment, remain popular due to their operational simplicity and low capital requirements. However, these techniques often yield lower recovery, require longer processing times, consume more energy and risk thermal or chemical degradation of sensitive proteins [10, 11, 82].

Modern technologies are increasingly adopted to enhance yield, preserve protein structure and reduce environmental impact. Ultrasound‐assisted extraction (UAE) has improved collagen yield and structural integrity in freshwater catfish skin [84], whereas microwave‐assisted hydrolysis further reduces processing time [77, 80]. Enzymatic hydrolysis, using proteases like pepsin or Alcalase, offers controlled collagen breakdown under milder conditions with higher selectivity and minimal chemical residues [76, 77]. Supercritical CO_2_‐acidified water has also been applied to striped catfish skin and bone, yielding around 37% collagen from skin and 8% from bone, highlighting its scalability potential.

The following sections discuss conventional and modern extraction technologies in terms of their mechanisms, applications, effectiveness, sustainability and suitability for recovering different products from freshwater fish by‐products.

### 3.1. Conventional Methods

Conventional extraction techniques remain widely used for valorising freshwater fish by‐products due to their accessibility, established protocols and relatively low operational costs. These methods primarily include acid/base treatments and solvent extraction, which can recover proteins, minerals, gelatine and hydroxyapatite [85]. Acidic treatments are commonly applied for recovering collagen, gelatine and mineral fractions from fish skin, scales and bones, thereby enhancing hydrolysis efficiency [10]. The acidic environment disrupts intermolecular cross‐links and increases collagen solubility, facilitating its release from connective tissues. Alkaline treatments are mainly used as pretreatment steps to remove noncollagenous proteins and residual lipids prior to gelatine or collagen extraction [11]. This process hydrolyses noncollagenous proteins and saponifies residual lipids, thereby improving the purity of collagen‐rich materials. In some protocols, sequential application of both acid and base treatments is carried out to optimise yield and purity, followed by neutralisation and drying to obtain functional powders [12].

In contrast, solvent‐based extraction uses organic solvents, such as isopropyl alcohol or ethanol, to dissolve nonpolar components and isolate proteins or lipids. The extraction mechanism relies on the differential solubility of lipids in organic solvents, allowing separation of hydrophobic compounds from proteinaceous tissues. This method is advantageous for obtaining relatively pure protein concentrates or removing unwanted lipids; however, it carries the risk of degrading sensitive amino acids, depending on the extraction temperature, duration and solvent concentration [79, 86]. Despite its efficiency, solvent recovery, toxicity concerns and the potential for protein denaturation are important limitations that must be considered when scaling up for food applications [22, 87].

Recent studies have also explored natural acid sources, such as *A. bilimbi,* as environmentally friendly alternatives to synthetic acids [10]. Similarly, base treatments such as NaOH solution have been used to remove non‐collagenous proteins or residual tissues from bones, thus aiding in mineral extraction [11]. Combined acid‐alkali approaches have also been utilised to produce nano‐calcium with improved bioavailability and reduced impurities [12].

In the context of protein recovery, producing gelatine from freshwater fish by‐products, such as skin, bones and scales, is a representative application of alkaline‐based extraction protocols. The standard process involves three main steps: (i) alkaline pretreatment to remove noncollagenous proteins and disrupt collagen cross‐links; (ii) extraction of collagen using hot water, typically by gradually increasing the temperature until it reaches 100°C; and (iii) filtration to eliminate insoluble contaminants that may reduce product quality [88]. Although this method does not always utilise both acid and alkali treatments, it demonstrates the usefulness of alkaline hydrolysis in facilitating the production of gelatine from collagen‐rich tissues.

Collagen, a structural protein rich in glycine, proline and hydroxyproline, is abundant in fish by‐products and serves as the precursor for gelatine. Its extraction may also be performed via acid‐soluble collagen (ASC) and pepsin‐soluble collagen (PSC) methods. ASC employs dilute organic acids under mild conditions, whereas PSC incorporates enzymatic digestion using pepsin to cleave peptide cross‐links, often resulting in improved yields [89]. These approaches have been studied in species such as catfish and tilapia, where the resulting collagen has been considered for applications in pharmaceutical, nutraceutical and cosmetic sectors [23].

In addition to protein extraction, conventional approaches have been used to recover minerals from bones and shells. For instance, calcite powders can be obtained from oyster shells through mechanochemical milling, followed by hydrothermal treatment to improve particle size distribution and thermal stability [90]. Other treatments, such as calcination and slaking, have also been used to convert bivalve shells into hydrated lime, offering an alternative to limestone‐derived products [90] These examples illustrate the continued relevance of conventional techniques in processing fish by‐products, particularly when adapted or optimised for specific applications. Table 3 provides an overview of conventional extraction methods for freshwater fish by‐products and representative application examples.

**Table 3:** Overview of conventional extraction methods for freshwater fish by‐products.

Sources	Extraction methods	Target compounds/products	Key parameters	Application example	Key highlights	References
Tilapia (*Oreochromis niloticus*) flesh	Isopropyl Alcohol solvent extraction	Fish protein concentrate	•Ratio of fish to solvent: 1:3 (*w*/*v*).	Dry protein ingredient for fortified food (Category C: protein < 60*%*; fat > 3*%*)	•Risk of amino acid degradation due to solvent, time or temperature.	Rieuwpassa et al. [79, 86]
			•Extraction time: 3 h.			
			•Drying: oven‐dried at 40°C for ~24 h.			
			•Post‐processing: Dried sediment is ground into powder and weighed to determine yield.			
Freshwater fish scales	Acid‐assisted hot water extraction using *Averhhoa bilimbi* (natural acid)	Gelatine	•Pretreatment: soaked in 3% bilimbi acid (1:4, 48 h).	Gelatine sheets or powder	•Eco‐friendly and natural acid source.	Syandri et al. [10]
			•Extraction: 70°C, 4 h (1:4 *w*/*v*).		•Long soaking time; potential variation in acid concentration.	
Armoured catfish (*Pterygoplichthys* spp.) bone	Thermal calcination	Hydroxyapatite	•Dried at 80°C (6 h).	Filler or reinforcement in chitosan‐based membranes	•Bioderived HA with potential in bone tissue engineering, environmental remediation.	López et al. [82]
			•Calcined at 1000°C (1.5 h, 6°C/min).			
			•Particle size 37–44 *μ*m.			
Pangas fish (*Pangasianodon hypophthalmus*) bone	Chemical and thermal treatment	Bone mineral powder	•0.2% NaOH boiling at 90°C for 30 min.	Mineral supplement	•NaOH treatment served as an organic cleaning agent to remove residual tissues from the bones and help minimise the fishy odour.	Antu et al. [11]
			•Autoclave at 121°C, 15 psi, 1 h.			
			•Oven drying at 80°C for 8 h.			
			•Pulverising and sieving (≤ 297 *μ*m).			
Tilapia, catfish	Acid‐alkali hydrolysis and neutralisation	Nano‐calcium powder	•Soak in 1 N HCl (1:5 *w*/*v*), stir 1 h, incubate 24 h at room temperature.	Nutraceuticals, calcium supplement	•Tilapia bone has a finer particle size of calcium than catfish (87.37 nm).	Kusumawati et al. [12]
			•Neutralise with 1 N NaOH, hydrolyse at 100°C for 60 min (×3 cycles).			
			•Neutralise to pH 6.9–7.1.			
			•Dry at 50°C for 15–18 h.			

### 3.2. Emerging Techniques

Emerging extraction technologies are increasingly explored as alternatives or complements to conventional methods for valorising freshwater fish by‐products. These techniques are aimed at enhancing extraction efficiency, minimising environmental impact and maintaining the functional integrity of target compounds. Among these, enzymatic hydrolysis, UAE, microwave‐assisted extraction (MAE) and supercritical fluid extraction (SFE) are the most widely studied methods.

Enzymatic hydrolysis is primarily used for producing FPH and collagen peptides by breaking down fish proteins. This method typically utilises proteolytic enzymes, such as Alcalase or Protana, under mild conditions to cleave peptide bonds, resulting in low‐molecular‐weight compounds with potential biological activities. Compared with chemical hydrolysis, enzymatic methods reduce by‐product contamination and minimise degradation of functional groups. For example, hydrolysates derived from Nile perch by‐products have demonstrated promising ACE‐inhibitory properties, although activities related to antidiabetic or neuroprotective effects were limited [76]. Furthermore, combining enzymatic hydrolysis with microwave‐assisted heating has been shown to enhance the degree of hydrolysis and improve protein functionality, including solubility and emulsifying capacity [77].

UAE is another emerging technique that improves mass transfer through acoustic cavitation, thereby accelerating the release of intracellular components. The cavitation bubbles generated during ultrasonication collapse near tissue surfaces, disrupting cellular structures and enhancing solvent penetration. UAE has been applied to extract collagen from catfish using dilute acetic acid, resulting in reduced lipid content and processing time compared with conventional acid extraction. The method also demonstrated a higher yield of protein‐rich collagen while operating at relatively low temperatures [84]. The UAE is often positioned as a supporting process to traditional solvent or acid‐based extractions to enhance recovery efficiency [91].

SFE, particularly with CO_2_, has attracted attention for its solvent‐free operation and adjustable parameters. Under supercritical conditions, CO_2_ exhibits gas‐like diffusivity and liquid‐like solvating properties, enabling efficient penetration into biological matrices and selective extraction of target compounds. In a previous study, the use of acidified water in combination with supercritical CO_2_ enabled the recovery of both collagen and gelatine from striped catfish skin and bone at relatively mild conditions [92]. Although this technique is energy‐intensive and requires specialised equipment, its potential for scalability and reducing solvent residue makes it a viable option for industrial applications.

MAE is also being explored to improve the recovery of oils and protein hydrolysate from fish by‐products. The mechanism involves dielectric heating, in which microwave energy rapidly heats polar molecules within the sample, thereby accelerating mass transfer and cellular disruption. Although MAE may not always yield the highest oil yields compared with methods such as wet rendering or Soxhlet extraction, it has been shown to preserve better polyunsaturated fatty acids (PUFAs) and other thermolabile compounds [80]. The method may therefore be better suited for applications where nutritional quality is prioritised over extraction efficiency. In the case of protein hydrolysates, microwave heating can also be combined with enzymatic hydrolysis to enhance functional properties, such as antioxidant activity or emulsifying ability [77].

Overall, although these emerging techniques offer environmental and functional benefits, their adoption at an industrial scale remains constrained by equipment cost, process optimisation requirements and product standardisation. A summary of selected applications is provided in Table 4.

**Table 4:** Overview of emerging extraction methods for freshwater fish by‐products.

Sources	Extraction methods	Target compounds/products	Key parameters	Application example	Key highlights	References
Nile perch (*Lates niloticus*) trimmings	Combined enzymatic treatments	Fish protein hydrolysate (FPH)	•Enzyme system: single‐stage Alcalase (endopeptidase) and Protana (exopeptidase)	Functional food and nutraceuticals (ACE‐inhibitory peptides)	•High protein yield.	Sapatinha et al. [76]
					•Production of low‐molecular‐weight peptides.	
			•50°C–60°C, pH 7.5–8.5, 1.5–3 h			
					•Potential antihypertensive application.	
					•Low levels of antidiabetic and anti‐Alzheimer activities.	
Striped catfish, catfish, tilapia	Hydrothermal extraction	Fish oil and gelatine	•Extraction at 121°C for 30 min in a batch autoclave	Raw material for supplements	•Extracted fish oil showed high content of omega‐9, omega‐6 and omega‐3 fatty acids.	Adnan et al. [53]
			•Fish‐to‐water ratio: 1:3 (*w*/*v*)			
			•Pressure controlled via vapour‐liquid equilibrium; thermocouple monitored; separation with separating funnel (oil‐rich top, gelatine‐rich bottom)			
					•No solvent use, but energy and water‐intensive.	
					•Potential degradation of heat‐sensitive compounds.	
			•Purified by centrifugation (6000 rpm, 30 min)			
			•Dried at 45°C for 10 h to obtain gelatin powder			
Catfish	Ultrasound‐assisted extraction (UAE) using 0.2 M acetic acid	Collagen	•Ultrasonic bath (150 min), ≤ 10°C; 2.5 M NaCl salting‐out	Collagen powder for functional food	•Removed up to 85.6% of lipid content and produced a potential source for collagen with high protein content.	Agustina et al. [84]
			•TRIS buffer neutralisation			
			•Centrifugation (6000 rpm, 15 min)			
			•Freeze‐drying at −54°C for 24 h			
					•Reduced extraction time.	
Striped catfish (skin and bone)	Supercritical carbon dioxide–acidified water extraction	Collagen and gelatin	•High‐pressure lab‐scale autoclave: 75 bar, 37°C, 24 h	Biomaterial engineering in healthcare	•Green solvent‐based method.	Phon et al. [92]
					•Suitable for industrial‐scale application.	
			•Solid/liquid ratio 1:25			
			•Freeze‐dry post‐treatment			
					•~37% yield (skin), ~8% (bone).	
Silver catfish (*Chrysichthys nigrodigitatus*), African sharptooth catfish (*Clarias gariepinus*), moustache catfish (*Synodontis membranaceous*)	Microwave (MW)–assisted wet rendering	Fish oil	•Microwave at 1200 W for 15 min	—	•Less effective than wet rendering in terms of oil yield.	Ayeloja et al. [80]
			•Water bath at 98°C for oil‐water separation			
					•Preserved better chemical and nutritional quality.	
Trout (*Oncorhynchus mykiss*) frames	Enzymatic hydrolysis using MW‐assisted heating	FPH	•Enzyme: Alcalase at 0.5%, 1.7%, 3.0% E:S (*w*/*v*)	—	•High degree of hydrolysis, protein solubility, emulsifying activity, foam capacity and antioxidant activity.	Nguyen et al. [77]
			•MW: 1200 W, 20% power, 50% duty cycle, 50°C–55°C, for 3, 5 and 15 min			
					•Best emulsifying results at 5 min (0.5% E:S); best foaming at 15 min (0.5% E:S).	

### 3.3. Comparative Discussion

In recent years, various extraction methods for obtaining bioactive compounds from freshwater fish by‐products have been compared in terms of yield, bioactivity retention and environmental sustainability. Among these, enzymatic hydrolysis has gained attention due to its mild reaction conditions, which help preserve the functional integrity of target compounds. This method is particularly effective in removing nonhelical regions of collagen, thereby improving its solubility and making it a preferred approach for collagen extraction from skin, scales and swim bladder residues of teleost fish [87]. However, a notable limitation is the risk of irreversible denaturation of the collagen structure during prolonged or high‐intensity enzymatic treatment. Moreover, exogenous enzymes can be applied to facilitate the oil extraction by hydrolysing the bonds between proteins and lipids, enabling the recovery of fish oil without significant degradation or solvent residues [93, 94]. Babajafari et al. [95] reported that although enzymatic extraction yielded slightly less oil compared with the chemical method, it produced oil with higher omega‐3 fatty acid content. Therefore, despite its lower extraction efficiency, the enzymatic method was considered a safer and more environmentally sustainable alternative.

In contrast, collagen nanotechnology has emerged as a novel strategy to enhance the functional properties of extracted collagen, especially for biomedical and pharmaceutical applications. Nano‐collagen has been explored for its potential use in bone grafting, vascular grafting, articular cartilage regeneration and wound healing [19]. Munahi et al. [70] demonstrated that nano‐collagen derived from catfish skin effectively accelerated burn wound healing in rabbits. Topical application of the nano‐collagen cream significantly reduced wound size, enhanced tissue contraction strength and promoted vascularisation and hair follicle regeneration compared with the untreated and skin‐grafted groups, indicating its strong potential as a functional product to improve skin repair and regeneration. Despite these promising findings, major technical and regulatory challenges remain, including the limited number of nano‐collagen formulations approved for commercial use and unsolved issues related to large‐scale production, stability and clinical efficacy [71].

From a sustainability perspective, enzymatic and assisted extraction methods (e.g., ultrasound‐ or microwave‐assisted hydrolysis) offer the advantage of lower environmental impact by reducing reliance on harsh chemicals typically used in conventional acid or alkaline extraction. Nevertheless, these advanced techniques often require higher initial investment and technical expertise, which may limit their scalability in low‐resource settings. Noman et al. [96] reported that ultrasound pretreatment enhanced the hydrolysis efficiency, yield, amino acid content and protein solubility of Chinese sturgeon (*Acipenser sinensis*) protein hydrolysates. In contrast, the combined ultrasonic‐microwave pretreatment with enzymatic hydrolysis achieved the highest antioxidant activity.

In terms of yield, hydrothermal and microwave‐assisted processing have also demonstrated higher extraction efficiencies in shorter processing times than conventional methods. However, bioactivity retention largely depends on thermostable compounds such as peptides and antioxidants [80, 97]. For instance, Adnan et al. [53] reported that hydrothermal extraction of striped catfish by‐products produced gelatine with acceptable gel strength and viscosity for industrial application, along with fish oil rich in omega‐3, ‐6 and ‐9 fatty acids. Although the overall yield was lower than that obtained using the acid‐based extraction method, it demonstrates the potential of hydrothermal for the simultaneous production of gelatine and fish oil.

Table 5 presents a further comparison of the advantages, limitations, yields and suitability of different extraction methods and target products for freshwater fish by‐products. The comparative analysis highlights that each extraction method for freshwater fish by‐products presents distinct trade‐offs in terms of yield, cost, environmental impact and scalability. Conventional methods such as acid and alkaline extraction are widely used due to their simplicity, scalability and relatively low cost; however, they raise environmental concerns about chemical use and waste generation and may affect protein integrity. Solvent‐based extraction improves lipid recovery but poses higher environmental and safety risks due to the use of organic solvents. Enzymatic hydrolysis stands out as an eco‐friendly and selective approach, offering high yields and improved functional properties, though its cost and longer processing times may limit industrial applications without optimisation. Emerging technologies such as ultrasound‐assisted, microwave‐assisted and supercritical CO_2_ extraction enhance efficiency, reduce processing time and improve extraction performance, but require higher capital investment and technical expertise. Overall, the selection of the extraction method depends on the targeted product, sustainability considerations and the balance between economic feasibility and environmental responsibility.

**Table 5:** Comparative analysis of extraction methods for freshwater fish by‐products.

Extraction method	By‐product	Target products	Yield expressed as	Reported yield (basis)	Advantage/limitations	Environmental impact; cost and scalability	References
Acid extraction	Skin (tra catfish, clown knifefish, tilapia)	Acid‐soluble collagen (ASC)	Freeze‐dried collagen per dry skin	12.5%–16.4% (dwb).	Simple protocol; widely applied/risk of collagen degradation; acid waste generated	Moderate to high (chemical use); low cost, highly scalable	Le et al. [69]
Acid‐assisted hot water extraction	Scales	Gelatin	Gry gelatin per dry scale	12.87%–18.29%.	Natural acidulant feasible/long soaking time; variable acid strength	Moderate; low cost, scalable	Syandri et al. [10]
Combined acid‐alkali hydrolysis	Bone (tilapia, catfish)	Nano‐calcium powder	Powder per dry bone	20%–50%.	Improved bioavailability; reduced impurities/multistep; neutralisation required	Moderate; low cost, scalable	Kusumawati et al. [12]
Alkaline and thermal treatment	Bone (pangas)	Calcium‐rich mineral powder	Powder per dry bone	Not reported.	Removes residual tissue and reduces fishy odour/protein denaturation at high pH	Moderate; low cost, scalable	Antu et al. [11]
Solvent extraction (isopropyl alcohol)	Flesh and trimmings (tilapia)	Fish protein concentrate (FPC)	Not reported as an extraction yield; product met FAO Category C (protein < 60*%*, fat > 3*%*)	Not reported.	Effective lipid removal; high purity/solvent toxicity; risk of amino acid degradation	High (organic solvents); moderate cost, scale‐up constrained	Rieuwpassa et al. [79, 86]
Enzymatic hydrolysis (Alcalase and Protana)	Trimmings (Nile perch)	FPH and bioactive peptides	Protein yield; degree of hydrolysis	Protein yields up to approximately 80%; degree of hydrolysis 34%–49%.	Mild conditions; selective; yields low‐molecular‐weight peptides/enzyme cost; overhydrolysis and bitterness risk	Low; moderate cost, scalable with optimisation	Sapatinha et al. [76]
Microwave‐assisted enzymatic hydrolysis	Frames (rainbow trout)	FPH	Degree of hydrolysis		Improves solubility, emulsifying activity and foaming capacity/non‐uniform heating	Moderate; moderate to high cost	Nguyen et al. [77]
Ultrasound‐assisted extraction (0.2‐M acetic acid)	Skin (*Pangasius* sp.)	Collagen	Collagen per dry skin	Not reported (study reports up to 85.6% lipid removal, which is not an extraction yield).	Reduced extraction time; low temperature; effective delipidation/equipment cost; limited standardisation	Low to moderate; moderate cost, scale‐up limited	Agustina et al. [84]
Microwave‐assisted extraction	Whole fish (three catfish species)	Fish oil	Oil per sample weight		Short processing time; better retention of polyunsaturated fatty acids/lower yield than wet rendering	Moderate; moderate to high cost, scale‐up challenging	Ayeloja et al. [80]
Supercritical CO_2_‐acidified water extraction	Skin (striped catfish)	Collagen and gelatin	Extract per dry skin	Approximately 37%.	Solvent‐free; minimal residue/high capital and operational cost	Low; high cost, industrially viable	Phon et al. [92]
	Bone (striped catfish)	Collagen and gelatin	Extract per dry bone	Approximately 8%.	As above	As above	Phon et al. [92]
Hydrothermal processing	Mixed by‐products (head, bone, skin and fins)	Gelatin	Gelatin per by‐product weight	2.51%–4.63%.	Simultaneous recovery of gelatin and oil; no solvent/lower yield than acid methods; heat‐sensitive compounds at risk	Low to moderate; moderate cost, scalable	Adnan et al. [53]
		Fish oil	Oil per by‐product weight	3.29%–13.56%.	As above	As above	Adnan et al. [53]

*Note:* Yields are reported as given in the original studies and are not directly comparable across rows, as they refer to different target products and are expressed on different bases (dry or wet weight of the starting by‐products). Entries are marked ‘not reported’ where the original study did not determine an extraction yield for the target product.

Abbreviations: dwb, dry weight basis; FPC, fish protein concentrate; FPH, fish protein hydrolysate; MAE, microwave‐assisted extraction.

Across freshwater species, several consistent trends emerge in the valorisation of fish by‐products. Collagen‐rich tissues such as skin, scales and bones across species, including catfish, tilapia and Nile perch, exhibit broadly comparable extraction behaviour, with enzymatic and assisted extraction methods consistently offering improved structural integrity and bioactivity retention compared with conventional acid‐alkali treatments. However, reported extraction yields vary across studies, reflecting differences in species‐specific tissue composition, pretreatment conditions, and analytical endpoints. A recurring gap in the literature is the lack of standardised protocols, which limits direct comparison of extraction efficiency and functional outcomes across freshwater species. Furthermore, although emerging technologies demonstrate promising improvements in quality and environmental performance, contradictions remain regarding their scalability and economic feasibility, highlighting the need for a harmonised evaluation framework [80, 87].

## 4. Stability and Storage of Extracted Compounds

The stability and storage of extracted bioactive compounds, including collagen, peptides, oils and mineral fractions from freshwater fish by‐products, are critical for ensuring their functional performance in food, nutraceutical and biomedical formulations. Following extraction, these compounds are vulnerable to multiple degradation pathways during processing, transportation and storage, which may result in loss of bioactivity, compromised functional properties, undesirable sensory changes and reduced shelf life [98]. The stability behaviours of extracted compounds are highly product‐dependent, as proteins, lipids and mineral fractions undergo different degradation mechanisms during storage.

**Figure 2:**
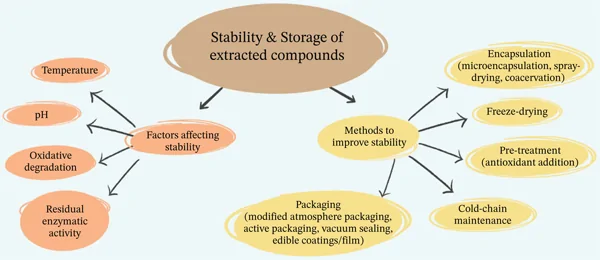
Factors influencing the stability of extracted compounds and strategies to enhance their stability.

In freshwater‐derived materials, instability arises primarily from chemical degradation (oxidation and hydrolysis) and biological activity (residual endogenous enzymes), with the dominant mechanism varying according to compound type and storage environment [99]. For example, protein‐based compounds such as collagen and peptides are particularly vulnerable to structural disruption, enzymatic cleavage and moisture‐induced hydrolysis [19, 100], whereas lipid fractions are highly prone to oxidation and hydrolysis‐induced rancidity [99]. Figure 2 provides an overview of factors affecting the stability of extracted compounds and potential methods to improve it. Because these mechanisms operate to different degrees in different products, the following subsections treat each product class separately rather than applying a single stability framework across the valorisation chain.

### 4.1. Principal Degradation Pathways

Four mechanisms account for most quality loss in compounds recovered from freshwater fish by‐products, and their relative importance differs markedly between product classes. Thermal denaturation disrupts hydrogen bonding and destabilises higher order protein structure, unfolding the collagen triple helix and reducing bioactivity [100]. Hydrolysis, whether chemical or enzymatic, cleaves peptide and ester bonds, lowering protein molecular weight and releasing free fatty acids from lipids. Oxidation proceeds through free‐radical chain reactions that generate hydroperoxides, which decompose into aldehydes, ketones and short‐chain volatile compounds responsible for rancidity and off‐flavours, and which also modify oxidation‐sensitive amino acid side chains such as methionine, cysteine and tyrosine [79, 86, 99]. Residual endogenous enzymes, including collagenase, peptidase and lipase, continue to act after extraction unless inactivated by heating, pH adjustment or chemical inhibitors and proteolytic activity can persist at refrigeration temperatures [99].

pH modulates all four pathways. Extreme acidic or alkaline conditions promote protein precipitation and denaturation, and prolonged exposure to acetic acid during extraction or storage lowers both the denaturation enthalpy and the denaturation temperature of collagen [19, 101]. Near‐neutral conditions of pH 6–7 generally favour retention of peptide structure and function, although microbial control then becomes the limiting consideration.

Which mechanism dominates, and therefore which stabilisation strategy is appropriate, is determined by the chemical nature of the product rather than by the species from which it was derived. The four main product classes are considered in turn below.

### 4.2. Collagen and Gelatine

For collagen and gelatine, thermal denaturation and moisture‐induced hydrolysis are the controlling mechanisms. Fish collagens generally exhibit lower thermal stability than mammalian collagens because of fewer intermolecular cross‐links and lower imino acid content [23, 102, 103]. Within fish, however, denaturation temperature varies considerably with habitat: ASC from freshwater species of the Mekong Delta reached a denaturation temperature of 33.09°C in clown knifefish, higher than values reported for cold‐water marine species [69]. Freshwater collagen is therefore somewhat more tolerant in storage than the figures commonly quoted for fish collagen would suggest, although it remains well below mammalian equivalents, and storage at temperatures approaching the denaturation threshold will promote unfolding and aggregation.

Residual acidity from extraction is a second consideration, as retained acetic acid lowers both denaturation temperature and enthalpy [19]; thorough neutralisation and washing before drying are therefore as important to stability as the storage conditions themselves. Because the remaining pathway is moisture‐dependent, the preferred stabilisation route is dehydration to a low water activity. Freeze‐drying is well suited to this product class, removing water at low temperature under vacuum and preserving triple‐helical structure, solubility and emulsifying properties more effectively than air‐drying or oven‐drying. Products are best held as dry powders or sheets in low‐permeability packaging under cool, dry conditions. Where collagen or gelatine is used in film form, the limiting factors shift to oxygen exposure and microbial growth, and modified atmosphere packaging or the incorporation of antioxidant or antimicrobial agents into the film matrix becomes more relevant than drying [104, 105].

### 4.3. Protein Hydrolysates and Peptides

Protein hydrolysates and peptides are structurally simpler than collagen and correspondingly less vulnerable to thermal denaturation, but more exposed to residual enzyme activity and oxidation. Because hydrolysis is itself the production step, incomplete enzyme inactivation allows proteolysis to continue during storage, and peptidases can further cleave short‐chain peptides, altering bioactivity, solubility and bitterness profile [78]. Complete inactivation by heat treatment at the end of hydrolysis is therefore the single most important stability intervention for this class. Oxidation of methionine, cysteine and tyrosine side chains represents the second pathway, reducing solubility and diminishing measured bioactivity [79, 86].

Encapsulation is the most effective stabilisation strategy for peptides, protecting against oxidative and enzymatic degradation while masking off‐flavours and improving handling. Spray‐drying and coacervation are the most commonly used techniques for peptide powders, and encapsulation of fish collagen hydrolysates in chitosan‐based microspheres has been reported to improve both stability and controlled release under simulated gastrointestinal conditions. Freeze‐drying is also appropriate where powder functionality must be preserved. Hydrolysate powders are hygroscopic and prone to caking, so moisture‐barrier packaging is required in addition to oxygen control [106–109].

### 4.4. Fish Oils

Oxidation is the dominant, and effectively rate‐limiting, mechanism for fish oils. The PUFAs that give these oils their nutritional value are also the source of their instability: Peroxidation generates hydroperoxides that decompose to aldehydes, ketones and short‐chain volatiles, producing rancidity and off‐flavours, and the reaction is accelerated by light, heat and exposure to air [99]. Hydrolytic rancidity is a secondary pathway, driven by residual lipase activity releasing free fatty acids.

Stabilisation therefore centres on excluding oxygen and interrupting radical propagation rather than on dehydration. Antioxidant addition, microencapsulation and oxygen‐restrictive packaging are the established approaches; microencapsulation has been used to improve the shelf life and bioavailability of fish oils [110, 111], with spray‐drying and complex coacervation the usual routes to a stable powder [107–109]. Modified atmosphere packaging, vacuum sealing and opaque containers limit exposure to oxygen and light, and cold‐chain maintenance slows propagation. Freeze‐drying, effective for the protein classes above, is not appropriate for lipid‐rich fractions, since the large surface area generated during drying accelerates oxidation unless antioxidants are incorporated. Freshwater fish oils contain lower PUFA concentrations than oily marine species [81], which moderates but does not remove the oxidation risk.

### 4.5. Mineral Fractions

Mineral products recovered from bone and scale, including bone flour, nano‐calcium and hydroxyapatite, behave differently from all of the above, and it is here that a single generalised treatment of stability is least defensible. The inorganic fraction is chemically stable and neither denatures nor oxidises under normal storage conditions. The factors limiting shelf life are physical and residual rather than chemical: moisture uptake causing caking and loss of flowability; particle agglomeration, which is of particular concern for nano‐scale products where the functional benefit depends on maintaining a fine particle size distribution [12]; and rancidity, discolouration or microbial growth arising from residual organic matter and lipid not removed during processing. The alkaline cleaning step applied to pangas bone by Antu et al. [11] is instructive in this respect, as removing residual tissue served both to purify the powder and to reduce fishy odour.

Stabilisation accordingly relies on effective removal of adhering organic material during processing, moisture control and packaging selected for water‐vapour barrier properties rather than oxygen exclusion. Chemical protection strategies such as antioxidant addition or encapsulation offer little benefit for this product class.

### 4.6. Matrix Interactions and Stability in Formulated Products

Once incorporated into a food, shelf life depends not only on the ingredient′s inherent stability but also on its interactions with the surrounding matrix, particularly pH, water activity and fat content. Antioxidant peptides may lose measurable efficacy in high‐fat matrices through interaction with the lipid phase, and hydrolysates can precipitate or develop off‐flavours in functional beverages over time unless encapsulated or flavour‐masked. Effective formulation therefore requires strategies tailored to both the ingredient and the product, including antioxidant pretreatment, appropriate carrier selection, control of water activity, and cold‐chain maintenance where required.

In summary, the stability of compounds recovered from freshwater fish by‐products cannot be described in general terms, because the dominant degradation pathway differs by product class: thermal denaturation and hydrolysis for collagen and gelatine; residual enzyme activity and oxidation for hydrolysates and peptides; lipid peroxidation for oils; and moisture uptake, particle agglomeration and residual organic matter for mineral fractions. Stabilisation strategies are correspondingly product‐specific, with freeze‐drying suited to protein fractions, encapsulation and oxygen exclusion to lipids and peptides, and moisture control to minerals. These relationships are summarised in Table 5. Matching the stabilisation approach to the individual product, rather than applying a single strategy across the valorisation chain, is essential to maintain bioactivity and extend shelf life.

## 5. Functional Food Applications

Considerable attention has been directed toward valorising fish processing wastes and by‐products as potential sources of functional food ingredients enriched with bioactive compounds. Such efforts are aimed at minimising industrial waste generation, enhancing environmental sustainability, stimulating economic value creation and formulating value‐added products beneficial to both human and animal health and overall well‐being [13]. Understanding the functional characteristics of bioactive compounds derived from freshwater fish by‐products is essential, particularly in determining how these components behave during food processing and how effectively they remain bioavailable once incorporated into the food matrix. Functional compounds obtained from fish by‐products possess important nutritional and physiological properties, contributing to the prevention of several health‐related disorders such as diabetes, cardiovascular diseases and other metabolic ailments [112, 113].

All fish species are recognised as valuable protein sources, as their protein content typically exceeds 13 g per 100 g of edible portion [114]. *Henicorhynchus siamensis*, a freshwater fish species, possesses a remarkable nutritional composition with high levels of protein (17.9 g/100 g), lipids (10.0 g/100 g) and ash (2.8 g/100 g). This nutrient‐rich profile makes it beneficial for undernourished individuals, promoting better health, supporting growth and contributing to overall metabolic well‐being [115]. Freshwater fish can be a valuable source of omega‐3 fatty acids, as the combined content of eicosapentaenoic acid (EPA) and DHA exceeds 80 mg per 100 g or 100 kJ, meeting the nutritional standards recommended by European guidelines [116]. Nevertheless, various fish species and their processing by‐products have significant pharmaceutical, nutraceutical, industrial and cosmeceutical potential, primarily because of the abundance of functional biomolecules, including collagen, enzymes, antioxidants, PUFAs and other bioactive constituents [117].

Fish by‐products, including the skin, muscle and bones, are rich in compounds with anti‐ageing, antimicrobial, wound‐healing and anticoagulant properties. These bioactive components play a vital role in protecting the skin against microbial invasion and other harmful agents [118]. Freshwater fish by‐products are increasingly recognised as valuable sources of functional food ingredients, as enzymatic hydrolysis yields protein hydrolysates rich in bioactive peptides that offer both nutritional and technological benefits. These FPHs exhibit notable antioxidant, antimicrobial and ACE‐inhibitory activities, making them promising candidates for incorporation into food products [64]. Peptides derived specifically from freshwater species such as tilapia have demonstrated clear functional food potential, such as the hydrolysates from tilapia, which showed significant antioxidant activity in cellular and animal models and exhibited additional anti‐inflammatory, anti‐diabetic and wound‐healing effects, thus validating their use in food or supplement applications [119].

Collagen and gelatine recovered from freshwater fish skins, scales and bones offer a compelling opportunity as functional food ingredients. For example, the extraction of collagen from the skins of commercial freshwater species such as red tilapia (*Oreochromis niloticus*), catfish (*C. gariepinus*) and pangasius (*Pangasius pangasius*) produced molecular − weight profiles > 80 kDa. It demonstrated good solubility and structure, suggesting strong potential for the use of their by‐products for other purposes [120]. Utilising collagen and gelatine in food formulations supports the development of health‐promoting products and enhances the sensory and structural attributes of processed foods [73]. Collagen and gelatine are also commonly used in the food industry because their unique structural, biochemical and physicochemical characteristics enhance nutritional and health benefits while improving the texture, stability and elasticity of various food products [72]. Gelatine obtained from fish or fish by‐products has attracted attention for its diverse biomedical functions. It can act as a dietary antihypertensive agent, promote wound repair and tissue regeneration, assist in managing osteoporosis and exert anticancer effects. Consequently, fish gelatine offers a viable alternative to traditional porcine and bovine gelatines used across various industrial sectors [74].

Long‐chain PUFAs from freshwater fish, including common species such as carp and catfish, can contribute valuable EPA and DHA to functional‐food formulations, although overall PUFA levels in many freshwater species tend to be lower and more variable than in oily marine fish but richer in amino acids [81]. A study by Ayeloja et al. [80] revealed that, regardless of the extraction technique employed, *Synodontis membranaceous* produced more oil than *Chrysichthys nigrodigitatus* and *C. gariepinus*. Additionally, the wet rendering process achieved the highest oil recovery, indicating its superiority over the other methods tested. Freshwater fish oils, extracted from by‐products of species such as tilapia, catfish and carp, contain a mix of long‐chain PUFAs, including EPA and DHA, albeit at lower levels than those in marine species [53]. For instance, this research investigates hydrothermal extraction as a novel valorisation approach for concurrently obtaining gelatine and fish oil from fish by‐products.

Freshwater fish by‐products, such as bones and scales, have attracted increasing attention as sustainable, nutrient‐rich materials for functional food applications, offering a valuable alternative source of calcium to traditional supplements [121]. Raw fish bones contain a remarkably high concentration of calcium, accounting for over 10% of their fat‐free dry weight, which is comparable to the calcium levels found in conventional sources like calcium citrate, calcium lactate, calcium gluconate and calcium acetate [65]. A study by Ashila et al. [122] used flour from red tilapia bones as a calcium source incorporated into doughnuts. Shrimp crackers enriched with nanocalcium derived from tilapia fish bone flour also represent a promising functional food innovation, offering a nutritious alternative that enhances calcium intake while maintaining desirable sensory qualities [123]. A recent study by Paramita et al. [124] demonstrated that incorporating catfish (*Clarias* sp.) bone flour into wheat mocaf dried noodles could effectively enhance the product′s mineral composition. Incremental additions of 0%–10% catfish bone flour notably elevated the calcium and phosphorus levels of the noodles, while maintaining acceptable texture and taste.

## 6. Prospects and Challenges

The valorisation of freshwater fish by‐products offers promising pathways for advancing aquaculture and fisheries toward circular bioeconomy models. Transforming nutrient‐rich residues, including skin, bones, scales, heads and viscera, into value‐added compounds enables the food industry to mitigate environmental impacts while enhancing economic profitability. The increasing global interest in functional, bioactive and sustainable food ingredients further drives initiatives to recover proteins, peptides, collagen, gelatine and oils from freshwater species [125]. Emerging innovations in green extraction technologies, integrated processing and quality standardisation are progressively facilitating the large‐scale implementation of fish by‐product utilisation, particularly in Southeast Asia, where freshwater aquaculture constitutes a major share of fish production.

### 6.1. Technological and Industrial Prospects

Emerging green technologies—such as enzymatic hydrolysis [76], UAE [84], microwave [80] and supercritical CO_2_ [92] —have demonstrated efficiency in recovering proteins and bioactive molecules with minimal environmental impact. Integrating multiple extraction strategies allows for the simultaneous production of gelatine, collagen hydrolysates and omega‐3–rich oils from species such as tilapia, pangasius and catfish [53]. Moreover, downstream innovations in fish by‐product valorisation, such as nano‐collagen fabrication and hydroxyapatite synthesis from bones, are paving the way for advanced biopolymers and nanocomposites with proven antimicrobial, bioactive and biocompatible properties for the biomedical, nutraceutical and cosmetic industries [84]. Encapsulation and freeze‐drying techniques are being optimised to enhance the stability, solubility and bioavailability of fish‐derived peptides and oils, allowing their incorporation into functional foods and beverages [106]. These technological advances, supported by automation and digitalisation, could enable scalable and traceable by‐product processing systems consistent with Industry 4.0 principles.

### 6.2. Economic and Sustainability Opportunities

Valorisation contributes directly to global sustainability goals by reducing organic waste, cutting carbon emissions and promoting responsible production (SDG 12.3 and 9.5). Economic circularity also extends to the substitution of conventional animal‐based raw materials. FPH and fish‐derived collagen, such as those sourced from fish scales, provide halal, ethical and allergen‐free alternatives to traditional bovine and porcine ingredients—meeting the specific dietary and market preferences found across Asia and the Middle East [75, 126]. Furthermore, regional initiatives promoting ‘zero‐waste aquaculture’ encourage collaborations between producers, researchers and policymakers to commercialise coproducts for food and non‐food industries.

Based on reported application examples and processing constraints, dietary supplements and powdered nutraceuticals have the highest commercial potential for freshwater fish by‐product ingredients, owing to minimal sensory challenges and simpler regulatory pathways. Functional beverages represent a promising but technically demanding category, requiring careful stabilisation and flavour masking to maintain bioactivity and consumer acceptance. Bakery and snack products offer additional opportunities, particularly for mineral‐rich and gelatin‐based ingredients, where thermal stability and texture modification can be leveraged without compromising product quality.

Despite their potential, the suitability of freshwater fish by‐product extraction methods depends largely on the targeted end product. Enzymatic hydrolysis is widely preferred for producing FPH and bioactive peptides due to its selectivity and ability to preserve bioactivity under mild processing conditions, although high enzyme costs and slow reaction rates may limit industrial scalability [76, 77]. UAE can improve yields and reduce solvent usage, yet scaling up requires high‐power equipment, increasing energy consumption [84]. MAE accelerates extraction and reduces solvent use, but uneven heating and high energy demands present technical barriers [77, 80]. Supercritical CO_2_ extraction is particularly suitable for recovering fish oils and lipid‐soluble bioactive compounds because it avoids solvent residues and oxidation, though the high capital and operational costs restrict wider industrial adoption [15, 64]. Therefore, selecting an appropriate extraction strategy should consider the intended product, processing cost, product stability and commercial application to ensure sustainable and economically viable utilisation of freshwater fish by‐products.

### 6.3. Scientific and Regulatory Challenges

Despite these advances, standardisation and regulatory harmonisation remain major challenges. Variations in raw material composition, freshness and processing conditions of freshwater fish by‐products lead to inconsistent yields and functional properties, as collagen yields can differ significantly depending on fish species, tissue source, environmental factors and extraction methods [98, 127]. Establishing analytical standards for purity, molecular weight and amino acid profiles of collagen and peptides is essential for quality assurance and international trade. Additionally, freshwater environments are vulnerable to contamination by heavy metals such as lead, chromium, cadmium and mercury, which can bioaccumulate in fish tissues and pose significant health risks to consumers. This necessitates stringent safety monitoring and traceability measures throughout the freshwater fish processing chain to safeguard public health and ensure product quality [25]. In many developing countries, fish by‐products are still legally classified as ‘waste’, which restricts their use in human food applications [128]. To fully harness their potential, regulatory reforms are essential to reclassify these materials as ‘aquatic co‐products’, supported by rigorous safety certification systems and aligned with international Codex Alimentarius guidelines to ensure safety and quality throughout the processing chain.

Beyond these structural and regulatory barriers, recent case studies illustrate how safety validation and consumer perception directly influence commercial translation. Murillo et al. [68] demonstrated that calcium‐rich catfish bone powder can be incorporated into breaded fish products without compromising sensory acceptance, with consumer purchase intent increasing when calcium fortification benefits were communicated, highlighting the importance of safety validation and regulatory compliance for mineral fortification. Brandão et al. [129] further showed that hydrolysed collagen from tilapia skin can be successfully commercialised as a novel functional product with high consumer acceptability and purchase intent, although regulatory approval for novelty ingredients and clear labelling remain essential. From a safety and regulatory perspective, Riyanto et al. [130] addressed halal authentication of catfish skin gelatine, demonstrating that feed traceability and DNA‐based verification are necessary to meet religious and regulatory requirements, underscoring that safety, certification and consumer trust are as critical as technological performance in enabling market adaptation of freshwater fish by‐product ingredients.

### 6.4. Scale‐Up and Market Implementation Challenges

Scaling laboratory‐scale processes to commercial production involves significant capital investment in extraction equipment, energy and wastewater management systems. Supercritical CO_2_ and ultrasound reactors, although effective, are costly and energy‐intensive, limiting access for small processors. Moreover, the high perishability of fish by‐products, irregular supply volumes and lack of cold‐chain logistics constrain continuous operation. The adoption of decentralised modular processing units near landing sites could alleviate some of these issues. Consumer acceptance also poses a persistent barrier. Although public awareness of sustainability is increasing, negative perceptions regarding ‘waste‐derived’ products can affect market growth [131]. Transparent labelling, education campaigns and product innovation—such as flavour‐neutral collagen beverages and biodegradable packaging—are vital to enhance consumer confidence. Cross‐sector collaboration between academia, industry and government agencies will be key to bridging the gap between pilot‐scale technologies and full commercial implementation.

## 7. Conclusion

Freshwater fish by‐products have significant potential for sustainable valorisation through the extraction of high‐value compounds, including collagen, gelatine, bioactive peptides and fish oils. This review highlights the growing emphasis on environmentally friendly extraction technologies that improve yield while preserving bioactivity. However, stability and storage of these compounds remain a challenge due to factors such as temperature, pH, oxidation and enzymatic degradation, which may affect their structural integrity and functional properties. Recent research demonstrates promising applications of freshwater‐derived compounds in functional foods, with proven health benefits including antioxidant, anti‐inflammatory and antihypertensive effects. To fully realise the potential of freshwater fish resources, further research should focus on optimising extraction conditions, improving compound stability and ensuring consumer acceptance. Integration of sustainable practices with innovative technologies will not only enhance the economic value of freshwater fish processing but also contribute meaningfully to food security and environmental conservation.

Compared with marine fish by‐products, freshwater fish by‐products generally exhibit lower salt content and reduced exposure to oxidative stress during processing, which can be advantageous for protein and collagen stability. However, freshwater species often show greater variability in tissue composition due to differences in farming practices, feed composition and environmental conditions, posing challenges for process standardisation. Marine by‐products, in contrast, benefit from more established valorisation pathways but are often associated with higher lipid oxidation and stronger odour profiles. These differences suggest that although extraction technologies developed for marine by‐products can inform freshwater applications, method optimisation must account for species‐specific composition and processing constraints unique to freshwater systems.

## Author Contributions

**Tan Yi-Li:** conceptualisation, data curation, writing – original draft. **Abu Bakar Asyrul-Izhar:** data curation, writing – original draft. **Nurul Izzah Khalid:** data curation, writing – original draft. **Rahmi Nurdiani:** writing – review & editing. **Nurul Huda:** writing – review & editing. **Yongyong Li:** writing – review & editing. **Mohammad Rashedi Ismail-Fitry:** conceptualisation, project administration, funding, supervision, writing – review & editing.

## Funding

This study was supported by the Geran Putra Inisiatif (KMD), Universiti Putra Malaysia (9764200) and the Department of Fisheries (DoF) Malaysia (6300578).

## Ethics Statement

No ethical approval was required for this study as it did not involve human participants.

## Conflicts of Interest

The authors declare no conflicts of interest.

## Data Availability

Data sharing is not applicable to this article as no datasets were generated or analysed during the current study.

## References

[1] FAO , The State of World Fisheries and Aquaculture 2024: Total Fisheries and Aquaculture Production. in the State of World Fisheries and Aquaculture 2024, 2024, FAO, 10.4060/CD0683EN.

[2] Islam J. , Yap E. E. S. , Krongpong L. , Toppe J. , and Peñarubia O. R. , Fish Waste Management -- an Assessment of the Potential Production and Uilization of Fish Silage in Bangladesh, Phillippines and Thailand, 2021, FAO, 10.4060/cb3694en.

[3] Islam M. J. and Peñarubia O. R. , Seafood Waste Management Status in Bangladesh and Potential for Silage Production, Sustainability. (2021) 13, no. 4, 10.3390/su13042372.

[4] Peñarubia O. , Toppe J. , Ahern M. , Ward A. , and Griffin M. , How Value Addition by Utilization of Tilapia Processing By-Products Can Improve Human Nutrition and Livelihood, Reviews in Aquaculture. (2023) 15, no. S1, 32–40, 10.1111/raq.12737.

[5] Tu E. and Tümerkan A. , The Novel Antioxidant Active Packaging Approach: A Combination of Aquafaba and Essential Oils to Prevent Lipid Oxidation in Fish, Biology and Life Sciences Forum. (2023) 26, 10.3390/FOODS2023-15159.

[6] AWARENET , Handbook for the Prevention and Minimization of Waste and Valorisation of By-Products in European Agro-Food Industries, 2004, Grow Programme.

[7] United Nations , Panel Underscores Untapped Potential of Aquatic Food Systems to Tackle Multiple Sustainable Development Goals, Including Ending Hunger, Protecting Ocean, 2025, https://press.un.org/en/2025/sea2229.doc.htm.

[8] Cabrita A. R. J. , Maia M. R. G. , Alves A. P. , Aires T. , Rosa A. , Almeida A. , Martins R. , and Fonseca A. J. M. , Protein Hydrolysate and Oil From Fish Waste Reveal Potential as Dog Food Ingredients, Frontiers in Veterinary Science. (2024) 11, 1372023, 10.3389/fvets.2024.1372023, 38711535. PMC11071340 38711535

[9] Bee S. L. and Hamid Z. A. A. , Hydroxyapatite Derived From Food Industry Bio-Wastes: Syntheses, Properties and Its Potential Multifunctional Applications, Ceramics International. (2020) 46, no. 11, 17149–17175, 10.1016/J.CERAMINT.2020.04.103.

[10] Syandri H. , Azrita A. , Elfrida E. , and Aryani N. , Biometric Indicators, Gelatin Yield, and Functional Properties of Fish Scale Waste From Three Species With Different Trophic Levels, International Journal of Food Science. (2025) 2025, no. 1, 6628788, 10.1155/ijfo/6628788. PMC12208754 40589460

[11] Antu M. A. R. , Ali M. S. , Ferdous M. J. , Ahmed M. T. , Ali M. R. , Suraiya S. , Pangestuti R. , and Haq M. , Recovery and Characterization of Calcium-Rich Mineral Powders Obtained From Fish and Shrimp Waste: A Smart Valorization of Waste to Treasure, Sustainability. (2024) 16, no. 14, 10.3390/su16146045.

[12] Kusumawati P. , Triwitono P. , Anggrahini S. , and Pranoto Y. , Nano-Calcium Powder Properties From Six Commercial Fish Bone Waste in Indonesia, Squalen Bulletin of Marine and Fisheries Postharvest and Biotechnology. (2022) 17, no. 1, 1–12, 10.15578/SQUALEN.601.

[13] Olaniran A. F. , Adeoye O. E. , Oyadeyi O. M. , Okonkwo C. E. , Erinle O. C. , Malomo A. A. , Iranloye Y. M. , Olaniran O. D. , and Faloye O. R. , Utilization and Application of Bioactive Compounds Generated From Fish Waste and by Product as Functional Food Ingredient: A Review, IOP Conference Series: Earth and Environmental Science. (2024) 1342, no. 1, 012014, 10.1088/1755-1315/1342/1/012014.

[14] Petchey O. L. and Gaston K. J. , Functional Diversity: Back to Basics and Looking Forward, Ecology Letters. (2006) 9, no. 6, 741–758, 10.1111/j.1461-0248.2006.00924.x, 16706917. 16706917

[15] Jiménez-Champi D. , Romero-Orejon F. L. , Muñoz A. M. , and Ramos-Escudero F. , The Revalorization of Fishery By-Products: Types, Bioactive Compounds, and Food Applications, International Journal of Food Science. (2024) 2024, no. 1, 6624083, 10.1155/2024/6624083, 39105167. PMC11300074 39105167

[16] Benkhoud H. , Baâti T. , Njim L. , Selmi S. , and Hosni K. , Antioxidant, Antidiabetic, and Antihyperlipidemic Activities of Wheat Flour-Based Chips Incorporated With Omega-3-Rich Fish Oil and Artichoke Powder, Journal of Food Biochemistry. (2021) 45, no. 3, e13297, 10.1111/jfbc.13297, 32515503. 32515503

[17] Yin T. , Khalifa I. , You J. , Peng L. , and Khoder R. M. , Fish Waste and By-Product as a Source of Calcium, Fish Waste to Valuable Products, 2024, Springer Nature, 10.1007/978-981-99-8593-7_10.

[18] Akhtar Hussain I. and Yasmin R. , Transforming Fish Waste for Economic and Environmental Gains, Scientia Review. (2025) 1, no. 1, 1–11.

[19] El Blidi O. , El Omari N. , Balahbib A. , Ghchime R. , El Menyiy N. , Ibrahimi A. , Kaddour K. B. , Bouyahya A. B. , Chokairi O. , and Barkiyou M. , Extraction Methods, Characterization and Biomedical Applications of Collagen: A Review, Biointerface Research in Applied Chemistry. (2021) 11, no. 5, 13587–13613, 10.33263/BRIAC115.1358713613.

[20] Salvatore L. , Gallo N. , Natali M. L. , Campa L. , Lunetti P. , Madaghiele M. , Blasi F. S. , Corallo A. , Capobianco L. , and Sannino A. , Marine Collagen and Its Derivatives: Versatile and Sustainable Bio-Resources for Healthcare, Materials Science and Engineering. (2020) 113, 110963, 10.1016/j.msec.2020.110963, 32487384. 32487384

[21] Nemati M. , Kamilah H. , Huda N. , and Ariffin F. , In Vitro Calcium Availability in Bakery Products Fortified With Tuna Bone Powder as a Natural Calcium Source, International Journal of Food Sciences and Nutrition. (2016) 67, no. 5, 535–540, 10.1080/09637486.2016.1179269, 27144766. 27144766

[22] Samant B. S. and Kaliappan R. , The Use of Surfactants in the Extraction of Active Ingredients From Natural Resources: A Comprehensive Review, RSC Advances. (2025) 15, no. 29, 23569–23587, 10.1039/D5RA02072G, 40630693. PMC12236305 40630693

[23] Zheng Y. , Li Y. , Ke C. , Gao X. , Liu Z. , and Chen J. , Comparison of Structural and Physicochemical Characteristics of Skin Collagen From Chum Salmon (Cold-Water Fish) and Nile Tilapia (Warm-Water Fish), Foods. (2024) 13, no. 8, 10.3390/foods13081213, 38672886. PMC11049058 38672886

[24] Ahmad N. I. , Mahiyuddin W. R. W. , Mohamad T. R. T. , Ling C. Y. , Daud S. F. , Hussein N. C. , Abdullah N. A. , Shaharudin R. , and Sulaiman L. H. , Fish Consumption Pattern Among Adults of Different Ethnics in Peninsular Malaysia, Food & Nutrition Research. (2016) 60, no. 1, 10.3402/FNR.V60.32697, 27534846. PMC4989178 27534846

[25] Hughes K. , The World′s Forgotten Fishes, 2021, https://www.panda.org/.

[26] Brummett R. E. , Clarias Catfish: Biology, Ecology Distribution and Biodiversity, Proceedings of a Workshop on the Development of a Genetic Improvement Program for African Catfish Clarias gariepinus. Worldfish Center Conference Proceedings, 2008, The World Fish Center.

[27] Ferosekhan S. , Giri A. K. , Sahoo S. K. , Radhakrishnan K. , Pillai B. R. , Shankar Giri S. , and Swain S. K. , Maternal Size on Reproductive Performance, Egg and Larval Quality in the Endangered Asian Catfish,*Clarias magur* , Aquaculture Research. (2021) 52, no. 11, 5168–5179, 10.1111/are.15385.

[28] Khan A. B. S. , Akhter M. , and Modak S. R. , Artificial Propagation of Asian Catfish, *Clarias batrachus* (Linnaeus, 1758) in Asia: A Review, Archives of Agriculture and Environmental Science. (2023) 8, no. 1, 92–96, 10.26832/24566632.2023.0801014.

[29] Kord A. , Benfares R. , Boudjema K. , Bourafa I. , Benaziza A. , Aliouche F. , Frai L. , and Touzouirt S. , By-Products From the Processing of Canned African Catfish (*Clarias gariepinus*): Potential for the Production of Fishmeal, Egyptian Journal of Aquatic Biology and Fisheries. (2025) 29, 2971–2985, 10.21608/ejabf.2025.449307.

[30] Herpandi H. , Manurung A. S. P. , Sudirman S. , and Widiastuti I. , Characteristics of Nanocalcium Derived From Snakehead Fish (*Channa striata*) Bone Meal Using Ultrasound-Assisted Extraction, Journal of Food Chemistry and Nanotechnology. (2025) 11, no. 2, 97–103, 10.17756/jfcn.2025-207.

[31] Sari S. F. and Rohma A. , Characterisation of Snakehead Fish, *Channa striata* (Bloch, 1793), Byproduct and Its Gelatin Properties, Asian Fisheries Science. (2024) 37, no. 3, 10.33997/j.afs.2024.37.3.003.

[32] Islami S. N. E. , Reza M. S. , Mansur M. A. , Hossain M. I. , Shikha F. H. , and Kamal M. , Rigor Index, Fillet Yield and Proximate Composition of Cultured Striped Catfish (*Pangasianodon hypophthalmus*) for Its Suitability in Processing Industries in Bangladesh, Journal of Fisheries. (2014) 2, no. 3, 157–162, 10.17017/j.fish.85.

[33] Khalid M. , Naeem M. , Azam S. M. , Ahmad N. , Kausar S. , Mustafa G. , Muhammad A. , Hira Q. , Irish A. , Shahid R. , Misbah F. , Ali Zafar K. , Saqlain I. , Parsa R. , Vijay L. , Najeeb U. R. , and Khursheed Z. , Morphometric and Length-Weight Relationship of the Nile Tilapia (*Oreochromis niloticus*) (Linnaeus, 1758) From Head Panjnad, Punjab, Pakistan, Egyptian Journal of Aquatic Biology & Fisheries. (2023) 27, no. 2, 25–33, 10.21608/ejabf.2023.289096.

[34] Relekar S. S. , Koli J. M. , Gore S. B. , Mohite A. S. , Desai A. S. , Wasave S. M. , Telvekar P. A. , and Pagarkar A. U. , Proximate and Amino Acid Composition of Freshwater Fish of Nagpur, Maharashtra, India, Indian Journal of Animal Research. (2024) 60, 144–149, 10.18805/ijar.b-5410.

[35] Ferguson C. , Kuhn D. , Murphy B. , O′Keefe S. , Phelps Q. , and Smith S. , Characterizing Biometrics and Nutrient Profiles of Fillet and Offal Components to Better Utilize Harvests of Invasive Carp in the US, Journal of Food Security. (2020) 8, no. 2, 52–65, 10.12691/jfs-8-2-3.

[36] Sahu B. B. , Raghunath M. R. , Meher P. K. , Senapati D. K. , Das P. C. , Mishra B. , Sahu A. K. , and Jayasankar P. , Comparative Studies on Carcass Characteristics of Marketable Size Farmed *Mrigalcirrhinus mrigala*, (Hamilton, 1822), and Silver Carp *Hypophthalmichthys molitrix*,(Val., 1844), Journal of Applied Ichthyology. (2014) 30, no. 1, 195–199, 10.1111/jai.12354.

[37] Zhu S. , Wu Z. , Zhang Y. , Chen W. , Li X. , He Y. , and Li J. , Age, Growth, and Mortality of Silver Carp *Hypophthalmichthys molitrix* (Valenciennes, 1844) in the Middle and Lower Reaches of the Pearl River, and Implications for Management and Conservation, Annales de Limnologie-International Journal of Limnology, 2021, 57, EDP Sciences, 10.1051/limn/2021019.

[38] Mawa Z. , Tanjin S. , Hossain M. Y. , Hasan M. R. , Rahman M. A. , Sabbir W. , Azad M. A. K. , Islam M. A. , Rahman M. A. , Jasmine S. , Basir Z. , Sarmin M. S. , and Ohtomi J. , Estimation of Relative Growth of *Anabas testudineus* Through Multiple Linear Dimensions, Egyptian Journal of Aquatic Biology and Fisheries. (2021) 25, no. 1, 935–952, 10.21608/ejabf.2021.153232.

[39] Herawati T. , Untung M. , Agung K. , Yustiati A. , and Suwartapradja S. , Characteristics of Meristics, Morphometrics, and Analysis of Cytochrome Oxidase Gene Fragment Subunit 1 (CO1) Hampala Barb (*Hampala* sp.) From Jatigede Reservoir, Sumedang, West Java, Indonesia, Journal of Survey in Fisheries Sciences. (2023) 10, no. 1, 2396–2406.

[40] Makmur S. , Arfiati D. , Bintoro G. , and Ekawati A. W. , Morphological, Meristic Characteristics and mtDNA Analysis of Hampala Fish (*Hampala macrolepidota* Kuhl & Van Hasselt 1823) From Ranau Lake, Indonesia, Journal of Biodiversity and Environmental Sciences (JBES). (2014) 5, no. 2, 447–455.

[41] Ibrahim N. H. M. , Yusoff N. F. M. , Zakeyuddin M. S. , Husin S. M. , Das S. K. , Nor S. M. , and Abdul Razak S. , Length-Weight Relationship and Condition Factor of Malaysian Mahseer (*Tor* sp.) From Selected Wild and Cultured Populations Across Peninsular Malaysia, Sains Malaysiana. (2025) 54, no. 3, 653–662, 10.17576/jsm-2025-5403-04.

[42] Kamarudin M. S. , Ramezani-Fard E. , Saad C. R. , and Harmin S. A. , Effects of Dietary Fish Oil Replacement by Various Vegetable Oils on Growth Performance, Body Composition and Fatty Acid Profile of Juvenile Malaysian Mahseer, *Tor tambroides* , Aquaculture Nutrition. (2012) 18, no. 5, 532–543, 10.1111/j.1365-2095.2011.00907.x.

[43] Srithongthum S. , Au H. L. , Amornsakun T. , Musikarun P. , Mok W. J. , Halid N. F. A. , Kawamura G. , and Lim L. S. , Reproductive Characteristics of the Pond-Farmed Sultan Fish (*Leptobarbus hoevenii*), Jurnal Ilmiah Perikanan Dan Kelautan. (2021) 13, no. 2, 171–180, 10.20473/jipk.v13i2.27264.

[44] Mohd Khairul Amri K. , Mohd Ekhwan T. , and Mushrifah I. , Analysis of *Leptobarbus hoevenii* in Control Environment at Natural Lakes, American Journal of Agricultural and Biological Science. (2013) 8, no. 2, 142–148, 10.3844/ajabssp.2013.142.148.

[45] Sahu B. B. , Barik N. K. , Routray P. , Agnibesh A. , Paikaray A. , Mohapatra S. , and Sundaray J. K. , Comparative Studies on Carcass Characteristics of Marketable Size Farmed Tilapia (*Oreochromis niloticus*) and Silver Barb (*Puntius gonionotus*), International Journal of Fisheries and Aquatic Studies. (2017) 5, no. 2, 6–9.

[46] Tamsil A. and Hasnidar H. , Reproductive Biology of Silver Barb, *Barbonymus gonionotus* (Bleeker, 1850), in Lake Tempe Indonesia, Iranian Journal of Fisheries Sciences. (2024) 23, no. 6, 877–892, 10.22092/ijfs.2024.131908.

[47] Murthy L. N. , Rao B. M. , Asha K. K. , and Prasad M. M. , Nutritional Composition, Product Development, Shelf-Life Evaluation and Quality Assessment of Pacu *Piaractus brachypomus* (Cuvier, 1818), Indian Journal of Fisheries. (2015) 62, 101–109.

[48] Ferraro V. , Cruz I. B. , Jorge R. F. , Malcata F. X. , Pintado M. E. , and Castro P. M. L. , Valorisation of Natural Extracts From Marine Source Focused on Marine By-Products: A Review, Food Research International. (2010) 43, no. 9, 2221–2233, 10.1016/J.FOODRES.2010.07.034.

[49] Muliajaya F. , Pratama F. , Herpndi , and Widiastuti I. , Chemical Characteristics of Scales, Skin, and Bones of Snakehead Fish (*Channa striata*) After Soaked With Sodium Hydroxide Solution, Journal of Smart Agriculture and Environmental Technology. (2024) 2, no. 1, 9–12, 10.60105/josaet.v2i1.42.

[50] Gill J. M. , Hussain S. M. , Ali S. , Ghafoor A. , Adrees M. , Nazish N. , Naeem A. , Naeem E. , Alshehri M. A. , and Rashid E. , Fish Waste Biorefinery: A Novel Approach to Promote Industrial Sustainability, Bioresource Technology. (2025) 419, 132050, 10.1016/J.BIORTECH.2025.132050, 39793671. 39793671

[51] FAO , The State of World Fisheries and Aquaculture 2020: Sustainability in Action, 2022, FAO, 10.4060/ca9229en.

[52] Ramakrishnan S. R. , Jeong C. R. , Park J. W. , Cho S. S. , and Kim S. J. , A Review on the Processing of Functional Proteins or Peptides Derived From Fish By-Products and their Industrial Applications, Heliyon. (2023) 9, no. 3, e14188, 10.1016/J.HELIYON.2023.E14188, 36938382. PMC10015205 36938382

[53] Adnan S. , Kresnowati M. T. A. P. , Marlina , and Bindar Y. , Sustainable Valorisation of Freshwater Fish By-Products to Gelatin and Fish Oil by Hydrothermal Extraction, International Journal of Food Science and Technology. (2024) 59, no. 11, 8224–8235, 10.1111/IJFS.17512.

[54] Corrêa S. S. , Gonçalves Oliveira G. , Coradini Franco M. , Gasparino E. , Feihrmann A. C. , Siemer S. , Dantas Filho J. V. , Cavali J. , de Vargas Schons S. , and Rodrigues de Souza M. L. , Quality of *Oreochromis niloticus* and *Cynoscion virescens* Fillets and Their By-Products in Flours Make for Inclusion in Instant Food Products, PloS One. (2023) 18, no. 2, e0279351, 10.1371/journal.pone.0279351, 36800330. PMC9937477 36800330

[55] Moraes P. S. , Engelmann J. I. , Igansi A. V. , Sant’Anna Cadaval Jr T. R. , and Antonio de Almeida Pinto L. , Nile Tilapia Industrialization Waste: Evaluation of the Yield, Quality and Cost of the Biodiesel Production Process, Journal of Cleaner Production. (2021) 287, 125041, 10.1016/j.jclepro.2020.125041.

[56] Franco M. L. R. S. , Franco N. P. , Gasparino E. , Dorado D. M. , Prado M. , and Vesco A. P. D. , Comparação das peles de tilápia do Nilo, pacu e tambaqui: Histologia, composição e resistência, Archivos de Zootecnia. (2013) 62, no. 237, 21–32, 10.4321/S0004-05922013000100003.

[57] Purnamayati L. , Dito B. S. , Dewi E. N. , and Suharto S. , Optimization of Tilapia (*Oreochromis niloticus*) Viscera Oil Extraction Using Response Surface Methodology, Food Research. (2023) 7, 12–20, 10.26656/fr.2017.7(S3).2.

[58] Sasongko H. , Zulpadly M. H. , and Farida Y. , An Evaluation of Potential Fatty Acids Nutrition in Snakehead Fish (*Channa striata*) Waste, Food Research. (2023) 7, no. 4, 30–35, 10.26656/fr.2017.7(4).285.

[59] Oktarlina R. Z. , Bahri S. , and Adjeng A. N. T. , Production and Characterization of Micro-Collagen From Carp Scales Waste (*Cyprinus carpio*), Research Journal of Pharmacy and Technology. (2022) 15, no. 5, 1995–2002, 10.52711/0974-360X.2022.00331.

[60] Wu J. , Kong L. , Zhang J. , and Chen W. , Extraction and Properties of Acid-Soluble Collagen and Pepsin-Soluble Collagen From Silver Carp (*Hypophthalmichthys molitrix*) Scales: Prerequisite Information for Fishery Processing Waste Reuse, Polish Journal of Environmental Studies. (2019) 28, no. 4, 2923–2930, 10.15244/pjoes/93742.

[61] Sanguino-Ortiz W. , Espinosa-Ruiz C. , Esteban Abad M. Á. , Román C. P. , and Hoyos-Concha J. L. , Effect of Fish Meal Substitution With Trout Viscera Protein Hydrolysate on the Innate Immune Response of Red Tilapia (*Oreochromis* spp), Fish Physiology and Biochemistry. (2025) 51, no. 2, 1–16, 10.1007/S10695-024-01444-0/METRICS. PMC11865115 40011259

[62] Vásquez P. , Sepúlveda C. T. , and Zapata J. E. , Functional Properties of Rainbow Trout (*Oncorhynchus mykiss*) Viscera Protein Hydrolysates, Biocatalysis and Agricultural Biotechnology. (2022) 39, 102268, 10.1016/j.bcab.2021.102268.

[63] Villamil O. , Váquiro H. , and Solanilla J. F. , Fish Viscera Protein Hydrolysates: Production, Potential Applications and Functional and Bioactive Properties, Food Chemistry. (2017) 224, 160–171, 10.1016/j.foodchem.2016.12.057, 28159251. 28159251

[64] Honrado A. , Miguel M. , Ardila P. , Beltrán J. A. , and Calanche J. B. , From Waste to Value: Fish Protein Hydrolysates as a Technological and Functional Ingredient in Human Nutrition, Foods. (2024) 13, no. 19, 10.3390/FOODS13193120, 39410155. PMC11482619 39410155

[65] Pérez A. , Ruz M. , García P. , Jiménez P. , Valencia P. , Ramírez C. , Pinto M. , Núñez S. M. , Park J. W. , and Almonacid S. , Nutritional Properties of Fish Bones: Potential Applications in the Food Industry, Food Reviews International. (2024) 40, no. 1, 79–91, 10.1080/87559129.2022.2153136.

[66] Gupta K. , Kutagulla T. , Shwethna K. , Prabhasankar P. , and Sachindra N. , Fortification of Extruded Food Products Using Fish Bone Powder as the Natural Calcium Source, 2018, 8th International Food Convention (IFCON).

[67] Kaliniak-Dziura A. , Skałecki P. , Florek M. , Kędzierska-Matysek M. , and Sobczak P. , Chemical Composition and Elements Concentration of Fillet, Spine and Bones of Common Carp (*Cyprinus carpio*) in Relation to Nutrient Requirements for Minerals, Animals. (2024) 14, no. 9, 10.3390/ani14091311, 38731315. PMC11083427 38731315

[68] Murillo S. , Ardoin R. , Watts E. , and Prinyawiwatkul W. , Effects of Catfish (*Ictalurus punctatus*) Bone Powder on Consumers′ Liking, Emotions, and Purchase Intent of Fried Catfish Strips, Foods. (2022) 11, no. 4, 10.3390/foods11040540, 35206021. PMC8871459 35206021

[69] Le T. M. T. , Nguyen V. M. , Tran T. T. , Takahashi K. , and Osako K. , Comparison of Acid-Soluble Collagen Characteristic From Three Important Freshwater Fish Skins in Mekong Delta Region, Vietnam, Vietnam. Journal of Food Biochemistry. (2020) 44, no. 9, 10.1111/JFBC.13397, 32713023. 32713023

[70] Munahi A. K. , Allban A. A. H. , Farman R. H. , and Towfik A. I. , Use of Nano-Collagen Creams Extracted From Catfish Skin in Treatment of Second-Degree Skin Burns in Rabbits, Macedonian Veterinary Review.(2025) 48, no. 2, 147–158, 10.2478/macvetrev-2025-0021.

[71] Rauta P. R. , Mohanta Y. K. , and Nayak D. , Nanotechnology in Biology and Medicine: Research Advancements and Future Perspectives, 2019, CRC Press, 10.1201/9780429259333.

[72] Ahmad M. I. , Li Y. , Pan J. , Liu F. , Dai H. , Fu Y. , Huang T. , Farooq S. , and Zhang H. , Collagen and Gelatin: Structure, Properties, and Applications in Food Industry, International Journal of Biological Macromolecules. (2024) 254, 128037, 10.1016/j.ijbiomac.2023.128037, 37963506. 37963506

[73] Zhang L. , Zhang S. , Song H. , and Li B. , Ingestion Of Collagen Hydrolysates Alleviates Skin Chronological Aging in an Aged Mouse Model by Increasing Collagen Synthesis, Food & Function. (2020) 11, no. 6, 5573–5580, 10.1039/D0FO00153H, 32520042. 32520042

[74] Usman M. , Sahar A. , Inam-Ur-Raheem M. , Rahman U. U. , Sameen A. , and Aadil R. M. , Gelatin Extraction From Fish Waste and Potential Applications in Food Sector, International Journal of Food Science and Technology. (2022) 57, no. 1, 154–163, 10.1111/ijfs.15286.

[75] Nikoo M. , Regenstein J. M. , and Yasemi M. , Protein Hydrolysates From Fishery Processing By-Products: Production, Characteristics, Food Applications, and Challenges, Foods. (2023) 12, no. 24, 10.3390/foods12244470, 38137273. PMC10743304 38137273

[76] Sapatinha M. , Camacho C. , Pais-Costa A. J. , Fernando A. L. , Marques A. , and Pires C. , Enzymatic Hydrolysis Systems Enhance the Efficiency and Biological Properties of Hydrolysates From Frozen Fish Processing Co-Products, Marine Drugs. (2025) 23, no. 1, 10.3390/md23010014, 39852515. PMC11766955 39852515

[77] Nguyen E. , Jones O. , Kim Y. H. B. , San Martin-Gonzalez F. , and Liceaga A. M. , Impact of Microwave-Assisted Enzymatic Hydrolysis on Functional and Antioxidant Properties of Rainbow Trout *Oncorhynchus mykiss* By-Products, Fisheries Science. (2017) 83, no. 2, 317–331, 10.1007/s12562-017-1067-3.

[78] Wang Y. , Zhao P. , Zhou Y. , Hu X. , and Xiong H. , From Bitter to Delicious: Properties and Uses of Microbial Aminopeptidases, World Journal of Microbiology and Biotechnology. (2023) 39, no. 3, 1–12, 10.1007/S11274-022-03501-3/METRICS. 36625962

[79] Rieuwpassa F. J. , Karimela E. J. , Chayono E. , Tomasoa A. M. , Ansar N. M. S. , Tanod W. A. , Nadia L. M. H. , Ramadhan W. , Ilhamdy A. F. , and Rieuwpassa F. , Extraction and Characterization of Fish Protein Concentrate From Tilapia (*Oreochromis niloticus*), Food Research. (2022) 6, no. 4, 92–99, 10.26656/fr.2017.6(4).528.

[80] Ayeloja A. A. , Jimoh W. A. , and Garuba A. O. , Nutritional Quality of Fish Oil Extracted From Selected Freshwater Fish Species, Food Chemistry Advances. (2024) 4, 100720, 10.1016/J.FOCHA.2024.100720.

[81] Ren H. T. , Jing Zhao X. , Huang Y. , and li Xiong J. , Combined Effect of Spirulina and Ferrous Fumarate on Growth Parameters, Pigmentation, Digestive Enzyme Activity, Antioxidant Enzyme Activity and Fatty Acids Composition of Yellow River Carp (*Cyprinus carpio*), Aquaculture Reports. (2021) 21, 100776, 10.1016/j.aqrep.2021.100776.

[82] López R. D. , Luna-Domínguez C. R. , Mendoza-Martínez A. M. , Şahin M. , Anzi B. S. , Cozza R. C. , and Luna-Domínguez J. H. , Development of Chitosan–Hydroxyapatite Membranes From Bone of Armoured Catfish (*Pterygoplichthys* spp.) for Applications in Guided Bone Regeneration (GBR), Processes. (2025) 13, no. 5, 10.3390/PR13051559.

[83] Aaddouz M. , Azzaoui K. , Sabbahi R. , Youssoufi M. H. , Yahyaoui M. I. , Asehraou A. , El Miz M. , Hammouti B. , Shityakov S. , Siaj M. , and Mejdoubi E. , Cheminformatics-Based Design and Synthesis of Hydroxyapatite/Collagen Nanocomposites for Biomedical Applications, Polymers. (2024) 16, no. 1, 10.3390/polym16010085, 38201750. PMC10780405 38201750

[84] Agustina M. , Patmawati P. , Mubarok S. , Sulmartiwi L. , Wulandari D. A. , Zai K. , Siva R. , Pujiastuti D. Y. , Nirmala D. , Werdani M. C. , and Moechthar O. , Effect of Ultrasonic Assisted Extraction With Ethanol for Removing Lipid on Catfish (*Pangasius* sp.) Skin as a Collagen Source and Its Characteristics, Jurnal Ilmiah Perikanan Dan Kelautan. (2024) 16, no. 1, 274–284, 10.20473/jipk.v16i1.46061.

[85] Hassan M. A. , Deepitha R. P. , Xavier K. A. M. , Gupta S. , Nayak B. B. , and Balange A. K. , Evaluation of the Properties of Spray Dried Visceral Protein Hydrolysate From *Pangasianodon hypophthalmus* (Sauvage, 1978) Extracted by Enzymatic and Chemical Methods, Waste and Biomass Valorization. (2019) 10, no. 9, 2547–2558, 10.1007/s12649-018-0302-1.

[86] Rieuwpassa F. J. , Karimela E. J. , and Lasaru D. C. , Karakterisasi Sifat Fungsional Konsentrat Protein Ikan Sunglir (*Elagatis bipinnulatus*), Jurnal Teknologi Perikanan Dan Kelautan. (2019) 9, no. 2, 177–183, 10.24319/jtpk.9.177-183.

[87] de Melo Oliveira V. , Assis C. R. , Costa B. D. , de Araújo Neri R. C. , Monte F. T. , da Costa Vasconcelos H. M. , França R. C. , Santos J. F. , de Souza Bezerra R. , and Porto A. L. , Physical, Biochemical, Densitometric and Spectroscopic Techniques for Characterization Collagen From Alternative Sources: A Review Based on the Sustainable Valorization of Aquatic By-Products, Journal of Molecular Structure. (2021) 1224, 129023, 10.1016/J.MOLSTRUC.2020.129023.

[88] Mad-Ali S. , Benjakul S. , Prodpran T. , and Maqsood S. , Interfacial Properties of Gelatin From Goat Skin as Influenced by Drying Methods, LWT - Food Science and Technology. (2016) 73, 102–107, 10.1016/j.lwt.2016.05.048. PMC5430197 28559624

[89] Song W. K. , Liu D. , Sun L. L. , Li B. F. , and Hou H. , Physicochemical and Biocompatibility Properties of Type I Collagen From the Skin of Nile Tilapia (*Oreochromis niloticus*) for Biomedical Applications, Marine Drugs. (2019) 17, no. 3, 10.3390/MD17030137, 30813606. PMC6471296 30813606

[90] Lu J. , Cong X. , Li Y. , Hao Y. , and Wang C. , Scalable Recycling of Oyster Shells Into High Purity Calcite Powders by the Mechanochemical and Hydrothermal Treatments, Journal of Cleaner Production. (2018) 172, 1978–1985, 10.1016/j.jclepro.2017.11.228.

[91] Lu W.-C. , Chiu C.-S. , Chan Y.-J. , Mulio A. T. , and Li P.-H. , Characterization and Biological Properties of Marine By-Product Collagen Through Ultrasound-Assisted Extraction, Aquaculture Reports. (2023) 29, 101514, 10.1016/j.aqrep.2023.101514.

[92] Phon S. , Pradana A. L. , and Thanasupsin S. P. , Recovery of Collagen/Gelatin From Fish Waste With Carbon Dioxide as a Green Solvent: An Optimization and Characterization, Recycling. (2023) 8, no. 2, 10.3390/RECYCLING8020030.

[93] Akimova D. , Kakimov A. , Suychinov A. , Urazbayev Z. , Zharykbasov Y. , Ibragimo N. , Bauyrzhanova A. , and Utegenova A. , Enzymatic Hydrolysis in Food Processing: Biotechnological Advancements, Applications, and Future Perspectives, Slovak Journal of Food Sciences. (2024) 18, 347–365, 10.5219/1962.

[94] Mamat M. N. I. , Abdul Rahman H. , Mohd Razali N. S. , Syed Hussain S. S. , Kasim K. F. , and Sofian-Seng N. S. , A Review on Fish Oil Extraction From Fish by-Product as Sustainable Practices and Resource Utilization in the Fish Processing Industry, Sains Malaysiana. (2025) 54, no. 1, 3627–3636, 10.17576/jsm-2025-5401-13.

[95] Babajafari S. , Moosavi-Nasab M. , Nasrpour S. , Golmakani M.-T. , and Nikaein F. , Comparison of Enzymatic Hydrolysis and Chemical Methods for Oil Extraction From Rainbow Trout (*Oncorhynchus mykiss*) Waste and Its Influence on Omega 3 Fatty Acid Profile, International Journal of Nutrition Sciences. (2017) 2, no. 2, 58–65.

[96] Noman A. , Qixing J. , Xu Y. , Abed S. M. , Obadi M. , Ali A. H. , al-Bukhaiti W. Q. , and Xia W. , Effects of Ultrasonic, Microwave, and Combined Ultrasonic-Microwave Pretreatments on the Enzymatic Hydrolysis Process and Protein Hydrolysate Properties Obtained From Chinese Sturgeon (*Acipenser sinensis*), Journal of Food Biochemistry. (2020) 44, no. 8, 10.1111/jfbc.13292, 32557735. 32557735

[97] Tian H. H. , Huang X. H. , and Qin L. , Insights Into Application Progress of Seafood Processing Technologies and Their Implications on Flavor: A Review, Critical Reviews in Food Science and Nutrition. (2024) 64, no. 33, 13259–13274, 10.1080/10408398.2023.2263893, 37788446. 37788446

[98] Jafari H. , Lista A. , Siekapen M. M. , Ghaffari-Bohlouli P. , Nie L. , Alimoradi H. , and Shavandi A. , Fish Collagen: Extraction, Characterization, and Applications for Biomaterials Engineering, Polymers. (2020) 12, no. 10, 10.3390/POLYM12102230, 32998331. PMC7601392 32998331

[99] Nemati M. , Shahosseini S. R. , and Ariaii P. , Review of Fish Protein Hydrolysates: Production Methods, Antioxidant and Antimicrobial Activity and Nanoencapsulation, Food Science and Biotechnology. (2024) 33, no. 8, 1789–1803, 10.1007/S10068-024-01554-8, 38752116. PMC11091024 38752116

[100] Nagai T. , Araki Y. , and Suzuki N. , Collagen of the Skin of Ocellate Puffer Fish (*Takifugu rubripes*), Food Chemistry. (2002) 78, no. 2, 173–177, 10.1016/S0308-8146(01)00396-X.

[101] Xiong Y. L. , Structure-Function Relationships of Muscle Proteins, Food Proteins and Their Applications, 2017, CRC Press, 341–392, 10.1201/9780203755617.

[102] Li Y. , Yang L. , Wu S. , Chen J. , and Lin H. , Structural, Functional, Rheological, and Biological Properties of the Swim Bladder Collagen Extracted From Grass Carp (*Ctenopharyngodon idella*), LWT - Food Science and Technology. (2022) 153, 112518, 10.1016/j.lwt.2021.112518.

[103] Zain N. M. , Saidin S. , and Sosiawan A. , Properties of Tilapia Collagen as a Biomaterial for Tissue Engineering: A Review, IOP Conference Series: Materials Science and Engineering. (2020) 932, no. 1, 012021, 10.1088/1757-899X/932/1/012021.

[104] Arul Raj J. S. J. S. , Kasi M. , Karuppiah P. , and Hirad A. H. , Green Packaging Solutions for Extending the Shelf Life of Fish Fillet: Development and Evaluation of Cinnamon Essential Oil-Infused Cassava Starch and Fish Gelatin Edible Films, ACS Omega. (2024) 9, no. 46, 45898–45910, 10.1021/ACSOMEGA.4C05249, 39583670. PMC11579729 39583670

[105] Huang R. , Yao A. , Yan Y. , Wang J. , Li Q. , Li K. , Tian Y. , Wang S. , and Wu J. , Development and Characterization of Multifunctional Fish Gelatin Composite Films Reinforced With *Ε*-Polylysine and Zinc Oxide Nanoparticles, EFood. (2024) 5, no. 4, 10.1002/EFD2.179.

[106] Hanifah A. , Kosasih W. , Ratnaningrum D. , Andriani D. , Putra H. E. , Yelliantty , and Priatni S. , Optimization of the Encapsulation of Lemuru Fish Protein Hydrolysate by Spray-Drying Using Response Surface Methodology, Food Technology and Biotechnology. (2025) 63, no. 1, 83–93, 10.17113/FTB.63.01.25.8626, 40322287. PMC12044295 40322287

[107] Peng J. , Zhang W. , Zi Y. , Shi C. , Kan G. , Gong H. , Wang X. , and Zhong J. , Fish Oil Encapsulation by Genipin-Crosslinked Complex Coacervation Between Gelatin and Different Anionic Polysaccharides, Food Hydrocolloids. (2024) 152, 109945, 10.1016/J.FOODHYD.2024.109945.

[108] Zhang W. , Chen L. , Bian Q. , Gong H. , Li L. , Wang Z. , Wang X. , and Zhong J. , Complex Coacervation of Low Methoxy Pectin With Three Types of Gelatins for the Encapsulation of Fish Oil, Food Chemistry. (2024) 460, 140567, 10.1016/J.FOODCHEM.2024.140567, 39059327. 39059327

[109] Zhang W. , Chen L. , Bian Q. , Wang Z. , Wang X. , and Zhong J. , Structures and Compositions of the Oil/Wall Interfaces and Wall Layers Affected the Properties of Spray-Dried Fish Oil Powders, Food Research International. (2025) 207, 116075, 10.1016/J.FOODRES.2025.116075, 40086961. 40086961

[110] Donmez D. , Limon J. , Russi J. P. , Relling A. E. , Riedl K. , Manubolu M. , and Campanella O. H. , Encapsulation of Fish Oil, a Triglyceride Rich in Polyunsaturated Fatty Acids, Within a Maillard Reacted Lecithin-Dextrose Matrix, Journal of Agriculture and Food Research. (2024) 18, 101283, 10.1016/J.JAFR.2024.101283.

[111] Liu J. , Mustapha W. A. W. , Zhang X. , and Li H. , Alkali-Induced Hydrolysis Facilitates the Encapsulation of Curcumin by Fish (*Cyprinus carpio* L.) Scale Gelatin, Foods. (2025) 14, no. 7, 10.3390/foods14071183, 40238373. PMC11989123 40238373

[112] Durazzo A. , Di Lena G. , Gabrielli P. , Santini A. , Lombardi-Boccia G. , and Lucarini M. , Nutrients and Bioactive Compounds in Seafood: Quantitative Literature Research Analysis, Fishes. (2022) 7, no. 3, 10.3390/fishes7030132.

[113] Kumari S. , Rath P. , Kumar A. S. H. , and Tiwari T. N. , Extraction and Characterization of Chitin and Chitosan From Fishery Waste by Chemical Method, Environmental Technology & Innovation. (2015) 3, 77–85, 10.1016/j.eti.2015.01.002.

[114] Bogard J. R. , Thilsted S. H. , Marks G. C. , Wahab M. A. , Hossain M. A. , Jakobsen J. , and Stangoulis J. , Nutrient Composition of Important Fish Species in Bangladesh and Potential Contribution to Recommended Nutrient Intakes, Journal of Food Composition and Analysis. (2015) 42, 120–133, 10.1016/j.jfca.2015.03.002.

[115] Sroy S. , Arnaud E. , Servent A. , In S. , and Avallone S. , Nutritional Benefits and Heavy Metal Contents of Freshwater Fish Species From Tonle Sap Lake With SAIN and LIM Nutritional Score, Journal of Food Composition and Analysis. (2021) 96, 103731, 10.1016/j.jfca.2020.103731.

[116] Bucchini L. , Nutrition and Health Claims in Europe: Oils & Fats Related Claims, Regulatory and Labeling Challenges, OCL. (2019) 26, 10.1051/ocl/2019041.

[117] Atef M. and Ojagh S. M. , Health Benefits and Food Applications of Bioactive Compounds From Fish Byproducts: A Review, Journal of Functional Foods. (2017) 35, 673–681, 10.1016/j.jff.2017.06.034.

[118] Noreen S. , Hashmi B. , Aja P. M. , and Atoki A. V. , Health Benefits of Fish and Fish By-Products—A Nutritional and Functional Perspective, Frontiers in Nutrition. (2025) 12, 1564315, 10.3389/fnut.2025.1564315, 40416371. PMC12098058 40416371

[119] Ng W. J. , Wong F. C. , Abd Manan F. , Chow Y. L. , Ooi A. L. , Ong M. K. , et al., Chai T. T. , Antioxidant Peptides and Protein Hydrolysates from Tilapia: Cellular and In Vivo Evidences for Human Health Benefits, Foods. (2024) 13, no. 18, 10.3390/foods13182945. PMC11431209 39335873

[120] Aminudin A. , Muhammad S. , Hamdan R. H. , Shaari R. , Nordin M. F. M. , Saufi R. A. , and Mei S. J. , Characterization of Collagen Extract From the Skins of Commercial Freshwater Fish, Jurnal Teknologi (Sciences & Engineering). (2015) 77, no. 33, 43–48, 10.11113/jt.v77.7003.

[121] Fatmawati S. , Istiqomah S. M. , Hasanah N. , Helan M. E. I. K. , Santoso M. , Nugraheni Z. V. , Jadid N. , Adi A. C. , and Rachmawati H. , Physico-Chemical Characterization of Natural Nano Calcium Extracted From Different Fish Bones in Catfish (*Clarias gariepinus*) and Snakehead Fish (*Channa striata*), Case Studies in Chemical and Environmental Engineering. (2025) 11, 101080, 10.1016/j.cscee.2024.101080.

[122] Ashila Y. , Pratama R. I. , Mulyani Y. , and Rostini I. , Fortification of Red Tilapia Bone Flour as a Source of Calcium on Doughnut Preference Level, Asian Journal of Fisheries and Aquatic Research. (2022) 18, no. 4, 17–26, 10.9734/ajfar/2022/v18i430448.

[123] Fatoni M. A. , Sumardianto S. , and Purnamayati L. , The Addition of Tilapia Bone Nanocalcium (*Oreochromis niloticus*) to the Physico-Chemical Characteristic of Shrimp Crackers, Jurnal Teknologi Hasil Pertanian. (2021) 14, no. 1, 1–10, 10.20961/jthp.v14i1.42545.

[124] Paramita B. L. , Sahubawa L. , and Ratnawati S. E. , Physicochemical Properties and Sensory Evaluation of Wheat-Mocaf-Based Dried Noodles Enriched With Catfish Bone Flour (*Clarias* sp.), Indonesian Food and Nutrition Progress. (2022) 18, no. 2, 60–66.

[125] Kendler S. , Sasidharan S. , and Rustad T. , Extraction of Proteinaceous Components and Biominerals From Cold Water Fish Fileting Side Streams: A Review, Frontiers in Sustainable Food Systems. (2024) 7, 1331113, 10.3389/fsufs.2023.1331113.

[126] Yasin , Is Collagen Halal? Exploring Its Benefits And Sources, 2025, https://www.yasingelatin.com/is-collagen-halal-exploring-its-benefits-and-sources.

[127] Chinh N. T. , Manh V. Q. , Trung V. Q. , Lam T. D. , Huynh M. D. , Tung N. Q. , Trinh N. D. , and Hoang T. , Characterization of Collagen Derived From Tropical Freshwater Carp Fish Scale Wastes and Its Amino Acid Sequence, Natural Product Communications. (2019) 14, no. 7, 1–12, 10.1177/1934578X19866288.

[128] Coppola D. , Lauritano C. , Palma Esposito F. , Riccio G. , Rizzo C. , and De Pascale D. , Fish Waste: From Problem to Valuable Resource, Marine Drugs. (2021) 19, no. 2, 10.3390/md19020116. PMC7923225 33669858

[129] Brandão T. M. , Mendes N. K. M. , Candeira W. K. D. A. , Borges J. M. , De Sousa P. H. M. , Da Silva R. A. , and Muratori M. C. S. , Molecular Caviar of Hydrolyzed Collagen From Tilapia Skin: Marketing and Sensory Perception, MOJ Food Processing & Technology. (2023) 11, no. 1, 43–49, 10.15406/mojfpt.2023.11.00278.

[130] Riyanto B. , Wahjuningrum D. , Ramadhan W. , and Al-Faruqi M. U. , Developing a Standard for Authenticating Halal Gelatine Catfish Skin: A Study on the Effect of Periodization Quarantine (Istihalah) on Gelatin Quality in Catfish Fed With Pig-Contaminated Feeds, Halal Studies and Society. (2023) 1, no. 1, 20–23, 10.29244/hass.1.1.20-23.

[131] Ou K. , Jiang A. L. , and Waller D. S. , From Scraps to Sweets: Perceptions of Food Healthfulness and the Acceptance of Upcycled Foods, Business Strategy and the Environment. (2026) 35, no. 1, 264–281, 10.1002/bse.70180.

